# An integrated single-cell atlas of blood immune cells in aging

**DOI:** 10.1038/s41514-024-00185-x

**Published:** 2024-11-29

**Authors:** Igor Filippov, Leif Schauser, Pärt Peterson

**Affiliations:** 1 QIAGEN Aarhus A/S, Aarhus, Denmark; 2 Institute of Biomedicine and Translational Medicine, University of Tartu, Tartu, Estonia

**Keywords:** Biomarkers, Senescence, Biomarkers

## Abstract

Recent advances in single-cell technologies have facilitated studies on age-related alterations in the immune system. However, previous studies have often employed different marker genes to annotate immune cell populations, making it challenging to compare results. In this study, we combined seven single-cell transcriptomic datasets, comprising more than a million cells from one hundred and three donors, to create a unified atlas of human peripheral blood mononuclear cells (PBMC) from both young and old individuals. Using a consistent set of marker genes for immune cell annotation, we standardized the classification of immune cells and assessed their prevalence in both age groups. The integrated dataset revealed several consistent trends related to aging, including a decline in *CD8*^*+*^ naive T cells and MAIT cells and an expansion of non-classical monocyte compartments. However, we observed significant variability in other cell types. Our analysis of the long non-coding RNA *MALAT1*^*hi*^ T cell population, previously implicated in age-related T cell exhaustion, showed that this population is highly heterogeneous with a mixture of naïve-like and memory-like cells. Despite substantial variation among the datasets when comparing gene expression between age groups, we identified a high-confidence signature of CD8^+^ naive T cell aging marked by an increased expression of pro-inflammatory genes. In conclusion, our study emphasizes the importance of standardizing existing single-cell datasets to enable the comprehensive examination of age-related cellular changes across multiple datasets.

## Introduction

Age is a significant risk factor for many immune-related diseases due to diminished immune responses and the onset of immunosenescence, leading to a compromised defense against infections and malignancies. It affects various biological pathways of the immune system, including innate and adaptive immune cell proportions and their functionality. For example, within the T cell compartment of peripheral blood mononuclear cells (PBMCs), *CD4*^*+*^ and *CD8*^*+*^ naïve T cell (Tn) frequencies decline, while the prevalence of effector memory T cells (Tem) rises with age^1^. Another consistent change occurs in monocytes, integral in immune defenses, which present increased proportions and decreased functionality over age^2^.

Single-cell RNA sequencing (scRNA-seq) is an indispensable technology for investigating phenotypic changes in different cell types of mixed populations with variable proportions. Recent publications have presented scRNA-seq datasets detailing the transcriptomic profiles of peripheral blood immune cells from both young and aged cohorts^1,3–8^. These datasets are important in identifying novel cell subsets associated with aging and measuring global transcriptomic changes, enabling the inference of transcriptional regulation patterns involved in immunosenescence. However, scRNA-seq analyses performed by different groups rely on disparate cell type definitions and marker genes, making direct comparisons between these studies challenging. Computational integration and re-analysis of the datasets can unveil novel insights, such as the cell type abundance and phenotype, difference in gene expression, and their associations with metadata attributes^9^.

We here present an integrated single-cell atlas of aging human PBMCs. Our combined dataset contains over a million high-quality gene expression profiles from seven cohorts of healthy young and old individuals. We reanalyzed the previously published CITE-seq PBMC data to assess the suitability of varying marker genes in distinguishing major T-cell subpopulations. We then performed a comprehensive cell type re-annotation with consistent marker genes across aging scRNA-seq studies. Using our integrated aging atlas, we searched for consistent gene expression signatures of immune aging across various cell types in healthy individuals from different cohorts.

## Results

### Data integration creates PBMC aging atlas

**Fig. 1:**
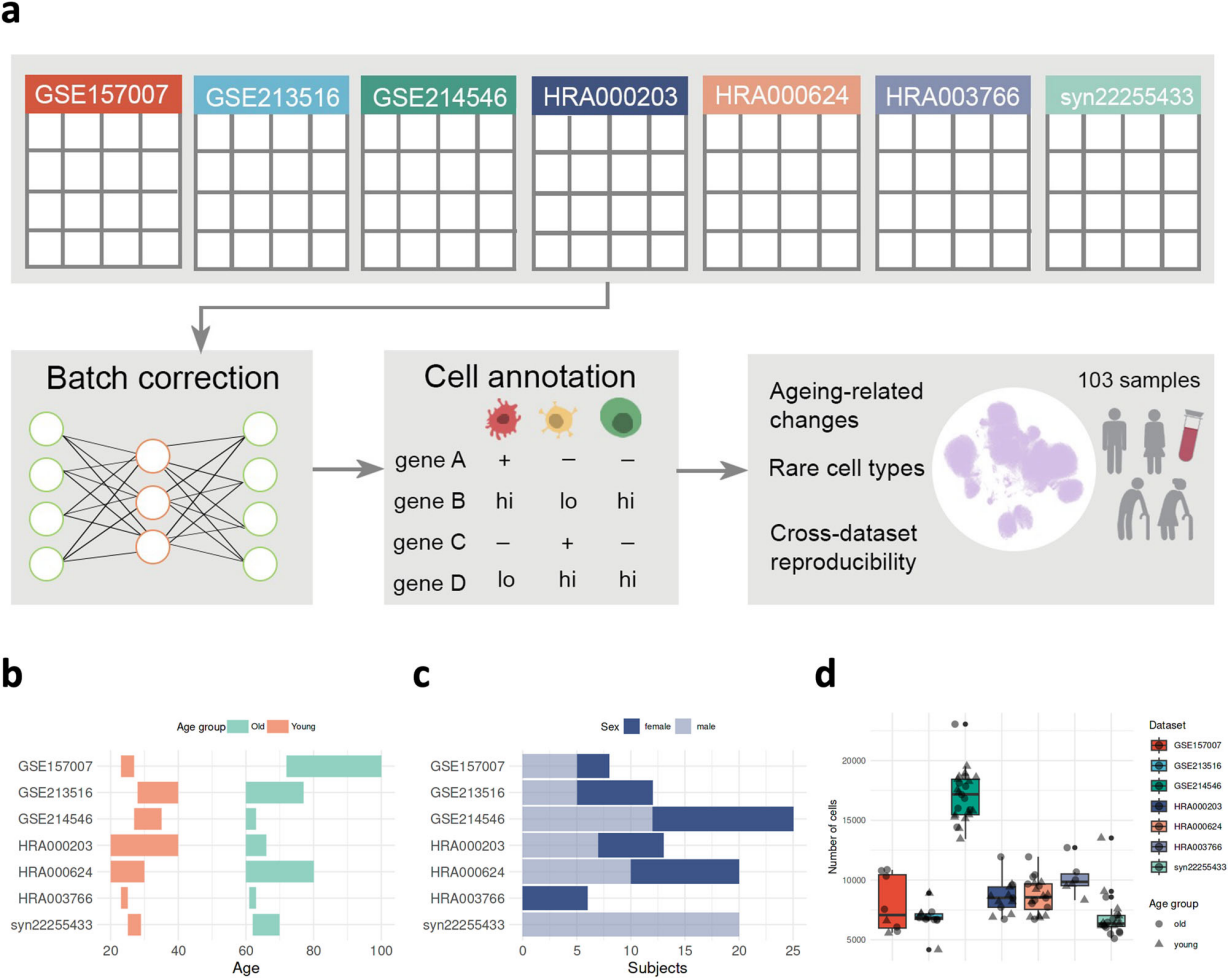
The PBMC ageing atlas study design. **a** The raw gene expression data and sample metadata were retrieved for seven aging human immune system studies. **b** Young and old age group definitions for each dataset. The horizontal bars represent the age range of each age group. **c** The donors’ sex distribution in GSE157007 (3 female, 5 male), GSE213516 (7 female, 5 male), GSE214546 (13 female, 12 male), HRA000203 (5 female, 7 male), HRA000624 (10 female, 10 male), HRA003766 (6 female), and syn22255433 (20 male). **d** The number of cells originating from each dataset in GSE157007 (2 young, 6 old), GSE213516 (5 young, 7 old), GSE214546 (16 young, 9 old), HRA000203 (7 young, 5 old), HRA000624 (10 young, 10 old), HRA003766 (3 young, 3 old), and syn22255433 (10 young, 10 old).

To create a comprehensive aging atlas, we collected data from seven recent studies where the PBMCs from young and old donors were subjected to the single-cell transcriptomic analysis using 10x Genomics technology. All donors included in the analysis were classified as healthy adults by the original authors (Fig. 1a). We downloaded the data from Luo et al. (GSE157007)^1^, Zhu et al. (GSE213516)^3^, Thomson et al. (GSE214546)^7^, Zheng et al. (HRA000203)^4^, Huang et al. (HRA000624)^5^, Li et al. (HRA003766)^8^, and Mogilenko et al. (syn22255433)^6^. The GSE213516, GSE214546, and HRA003766 were generated using the 3' version of the 10x Genomics scRNA-seq protocol, while four other datasets were made with the 5' single-cell sequencing chemistry. In total, we studied the gene expression profiles of 53 young (22 female, 31 male) and 50 aged (22 female, 28 male) individuals (Table S1). The original age group definitions differed between the studies; therefore, we applied additional sample exclusion criteria. For the young group, we excluded samples from donors older than 40; for the old group, we retained samples from donors over 60 (Fig. 1b**;** Table S1). In their sex distribution, all datasets included both male and female donors, except syn22255433, which consisted exclusively of male samples, and HRA003766, which consisted exclusively of female samples (Fig. 1c). The average number of cells detected after the analysis differed between the datasets, and there was higher cell number variability in GSE157007 compared to others (Fig. 1d).

We first combined the data from all the studies into one gene expression object with 14,461 gene profiles for 1,115,136 cells. After removing cell doublets, low-quality, and *CD45*^*-*^ cells, the resulting filtered dataset contained transcriptomic measurements for 1,047,793 high-quality PBMCs (Fig. S1a–g). We investigated whether there was a batch effect that would affect data interpretation between the datasets included in our analysis. To this end, we performed a standard scRNA-seq processing workflow, which included normalization and dimensionality reduction. After examining the UMAP plots with cells colored by the study of the origin, we noticed that cells originating from each dataset formed distinct communities, indicating a severe batch effect (Fig. S2a). We, therefore, applied the scVI software^10^ to integrate all the datasets and to reduce the dimensionality of data for unified clustering. The scVI model implements a flexible generative model based on variation inference, creating a low-dimensional data representation for downstream analyses. We trained the scVI model on the combined dataset, which resulted in a significantly reduced dimensionality, and after the batch effect correction, the cells of all seven datasets showed correct clustering into each main PBMC population (Fig. S2b).

### PBMC aging atlas enables comprehensive cell type annotation

**Fig. 2:**
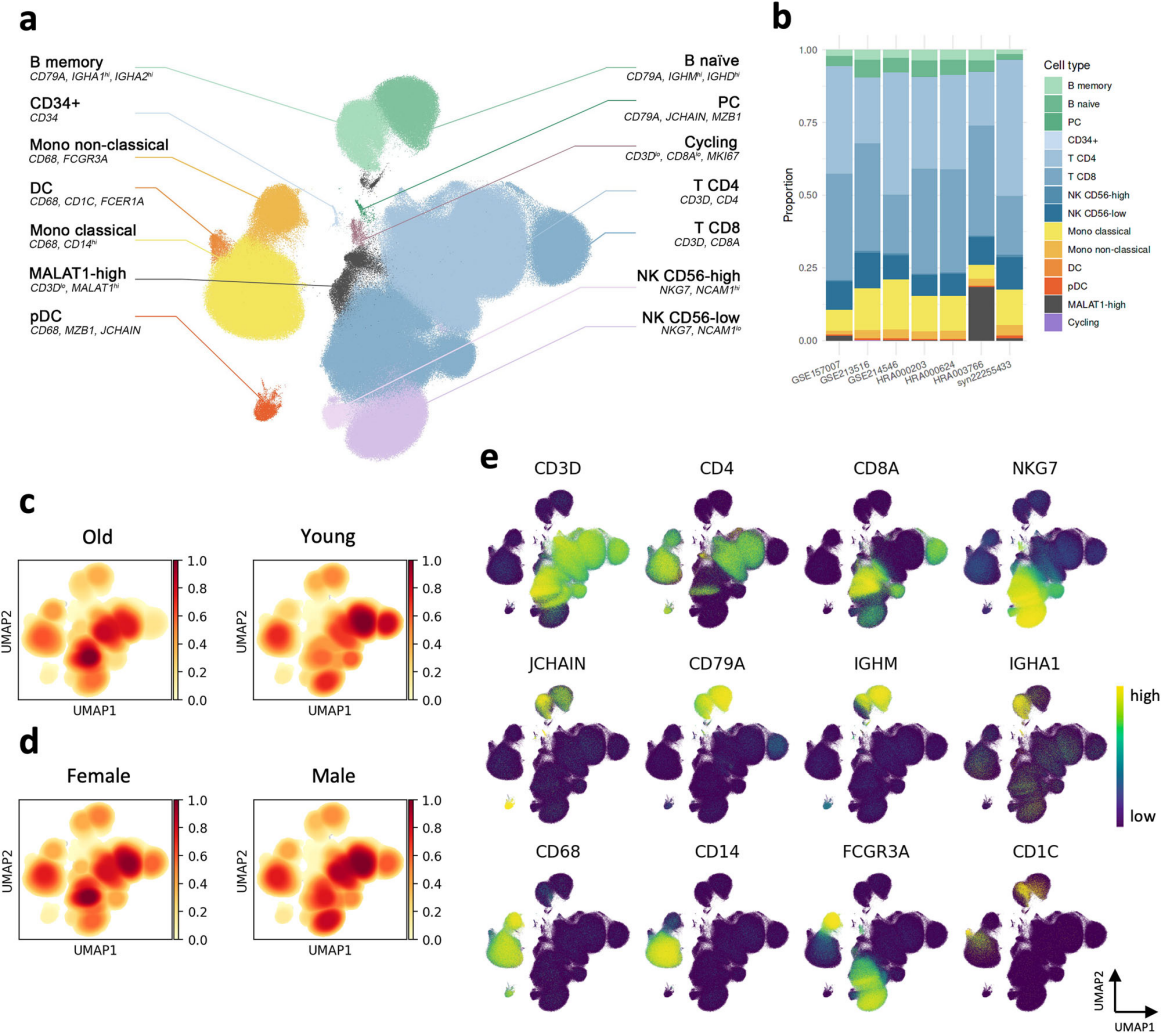
Integrated PBMC ageing atlas reveals heterogeneity between ageing studies. **a** UMAP representation of the 1,047,793 PBMCs from young and old subjects identified by integrating seven scRNA-seq datasets. **b** Bar plots showing proportions of the major PBMC subpopulations in each dataset. **c** Estimation of young (*n* = 53) and old (*n* = 50) groups density in the UMAP space. **d** Estimation of female (*n* = 44) and male (*n* = 59) groups density in the UMAP space. **e** Gene expression of selected marker genes used to identify the major PBMC populations overlaid on the UMAP representation.

In contrast to the flow cytometry of PBMCs that use well-established cell surface markers to distinguish immune cell subsets, no consensus on transcriptomic annotation markers exists in the scRNA-seq field, partly because of the low cell numbers of subpopulations and their high heterogeneity. Our large-scale integrated PBMC dataset allowed us to harmonize cell type definitions across the studies using a uniform set of annotation markers. We, therefore, performed a full re-annotation of the combined PBMC dataset based on known marker genes (Fig. 2a, e; Fig. S3a,b). Starting from the B cells (*CD79A*), we identified clusters of naïve (*IGMH, IGHD*) and memory (*IGHA1, IGHA2*) B cells. In addition, we observed a small population of plasma cells (PC; *CD79A*, *JCHAIN*, *MZB1*). Among the myeloid cells, we identified classical (*CD68*, *CD14*) and non-classical (*CD68, FCGR3A*) monocytes, as well as dendritic cells (DC; *CD68*, *CD1C*, *FCER1A*). We also annotated a cluster of plasmacytoid dendritic cells (pDC; *CD68*, *MZB1*, *JCHAIN*). The natural killer (NK) cell compartment was represented by *CD56*^*hi*^ and *CD56*^*lo*^ NK cells. Among the T cells, we identified large populations of CD4^+^ and CD8^+^ T cells. Other cell populations included *CD34*^+^ cells, likely representing circulating hematopoietic progenitors, cycling cells (*MKI67*), and a subset of cells with high expression of long non-coding RNA (lncRNA) *MALAT1*. The analysis of integrated populations showed the *MALAT1*^*hi*^ cells to have the lowest number of UMIs per cell and the highest percentages of mitochondrial genes (Fig. S3c, d). In addition, we subset and re-analyzed the B cells to further characterize the 77,378 B cells from seven studies and identified the atypical B cells (ABCs) subset in addition to naive and memory B cells (Fig. S4a, b).

With the consistent cell type markers across all datasets included in our age-related PBMC atlas, we inquired whether the annotated cell subsets were equally abundant in all studies. We observed pronounced differences between the seven datasets in major immune compartments (Fig. 2b, Table S2a). The proportion of CD8^+^ T cells was lower in GSE214546 and syn22255433, while syn22255433 and GSE157007 had fewer B cells than other datasets. In addition, GSE157007 and HRA003766 had fewer monocytes compared to the five other datasets. Interestingly, HRA000203 and HRA000624 had only minor differences in cell type proportions despite the distinct age group definitions in these two studies. Finally, we noticed that a population of *MALAT1*^*hi*^ cells was unevenly distributed among the studies and HRA003766 had the highest abundance of these cells. We tested if some donor samples contributed a disproportional number of cells to a given PBMC subtype in each dataset. Indeed, we found that some donors contributed significantly more cells in datasets with fewer samples, like GSE157007 and HRA000766 (Fig. S5a, b). However, all of the cell types contained cells originating from multiple datasets and donors. We performed an integration workflow using Harmony software^11^ to validate our results, demonstrating a high correlation between cell type proportions between both batch effect correction tools (Fig. S6a–d). Therefore, the resulting cell type communities in our atlas are robust to the choice of computational pipeline and provide a comprehensive overview of cellular landscapes in aging.

Next, we investigated the effects of age and biological sex on the distribution of PBMCs by performing Gaussian kernel density estimation for each cell and overlaying the results on UMAP (Fig. 2c, d). This analysis revealed differences between age and sex groups across various populations, including classical monocytes and NK cells. However, the most pronounced variations were observed in the subpopulations of CD8^+^ T cells.

### Defining T cell markers for Tn and Tcm populations

We next focused on the T cell compartment in the consolidated PBMC dataset because of its most significant shift among immune cells over age. The CD4^+^ and CD8^+^ T cell subsets undergo major rearrangements during aging with the decline of Tn numbers and an increase in Tem populations. Single-cell transcriptome studies often define cell types using diverse marker genes, which may confuse proper cell identification and hinder between-study comparisons.

One of the challenges in T cell single-cell annotations is separating CD4^+^ and CD8^+^ Tn and central memory (Tcm) T cells because of their overall similarity in gene expression profiles. For example, Zheng and colleagues annotated CD4^+^ Tn and Tcm cells by their *CCR7* and *CD69* expression; however, they could not separate the CD8^+^ Tcm population^4^. In contrast, Luo et al. identified CD4^+^ and CD8^+^ Tcm cells but could not distinguish between CD4^+^ and CD8^+^ Tn cells^1^. To overcome these disparities in Tn and Tcm and other T cell classifications, we searched for the transcriptomic markers that could be used to separate T cell populations reliably. To this end, we downloaded a CITE-seq reference dataset of 73,060 T cells from eight donors who participated in an HIV vaccine trial^12,13^. The surface protein antibody panel included *CD45RA* and *CD45RO* marker detection, commonly used to distinguish Tn and Tcm subsets in flow cytometry.

To reliably separate major CD4^+^ and CD8^+^ T cell subsets, including Tn and Tcm, we first applied the totalVI probabilistic model^14^, which uses the transcriptomic and surface protein measurements to create a combined multimodal data representation for dimensionality reduction and clustering. After performing the UMAP analysis, we observed that the cell communities corresponded to their original cell type annotations (Fig. S7a, b). We found CD4^+^ and CD8^+^ Tn cells to express naïve cell markers *LEF1*, *TCF7*, *CCR7*, and *SELL* at higher levels than Tcm. More distinctly, the *LRRN3* expression, expressed in CD4^+^ and CD8^+^ Tn cells, was present in only small fractions of Tcm cells, whereas the *CD69* gene was expressed at higher levels in Tcm cells. Furthermore, *PASK* was expressed more highly in CD8^+^ Tn cells compared to CD4^+^ Tn. We also observed that *KLRG1*, *GZMK*, *GNLY*, and *NKG7* were more abundantly expressed in CD8^+^ Tem cells compared to Tcm population. The transcription factors *EOMES* and *TBX21* were associated with the T cell cytotoxicity as they were present in CD4^+^ CTLs and CD8^+^ Tem cells.

In conclusion, our marker gene re-analysis showed that for the separation of Tn and Tcm cells, CD4^+^ Tn cells could be identified by high expression levels of *LEF1, TCF7, CCR7, SELL* and *LRRN3*. Although the CD4^+^ Tcm cells share these markers, they have low *LRRN3* expression and are also positive for the *PASK* gene. The same markers can be used for CD8^+^ Tn and Tcm annotations, except for PASK, which is more highly expressed in CD8^+^ Tn population. Both CD4^+^ and CD8^+^ Tcm can be characterized by *CD69* marker.

### T cell re-annotation reveals between-study differences

**Fig. 3:**
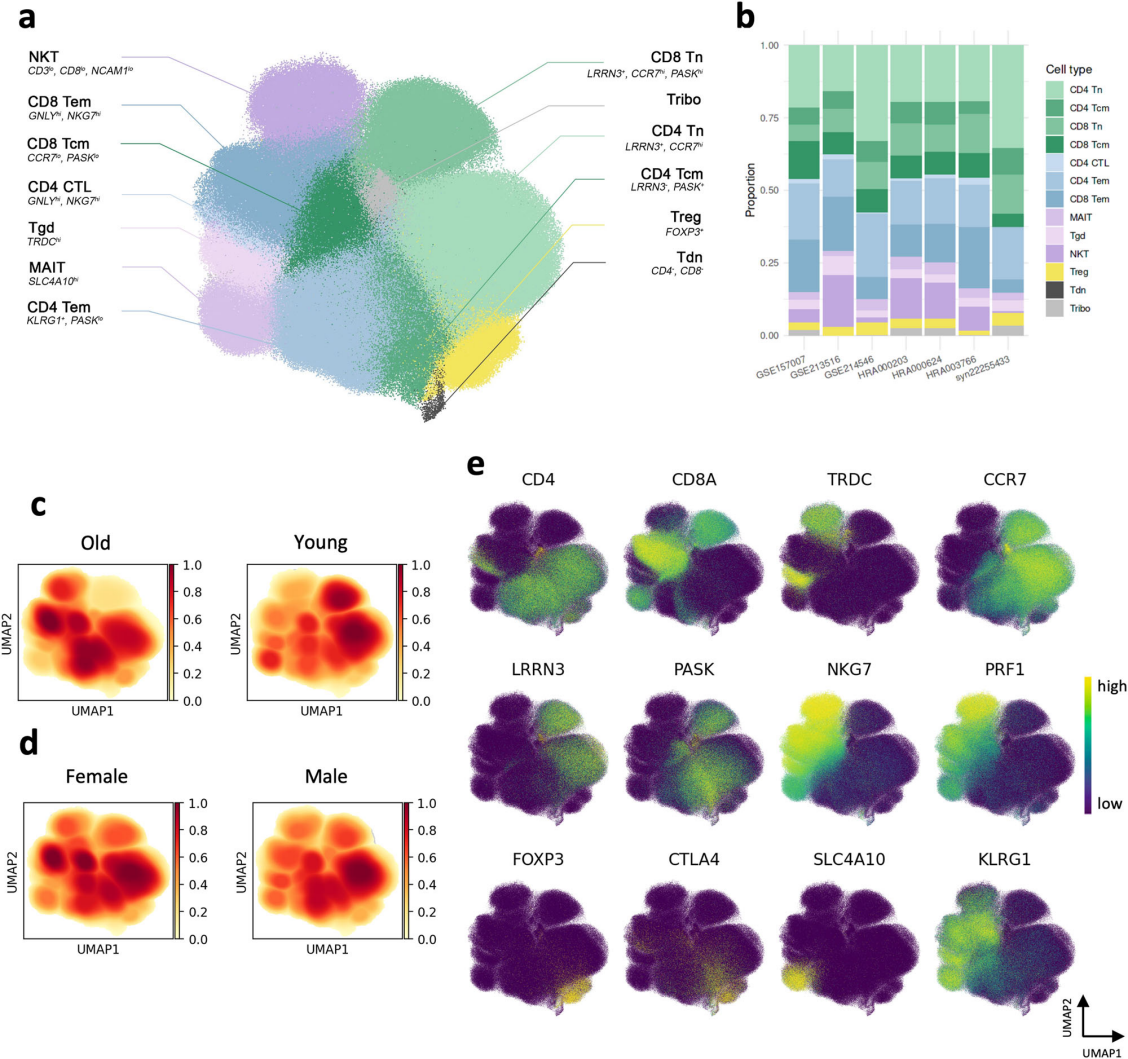
Integrated analysis of young and old T cells. **a** UMAP representation of the 672,765 T cells from young and old subjects identified by reanalysis of T cell subsets. **b** Bar plots showing proportions of T subpopulations in each dataset. **c** Estimation of young (*n* = 53) and old (*n* = 50) groups density in the UMAP space. **d** Estimation of female (*n* = 44) and male (*n* = 59) groups density in the UMAP space. **e** Gene expression of selected marker genes used to identify the T cell subsets overlaid on the UMAP representation.

With this robust set of T cell markers, we reannotated T cell subsets as the largest populations in our aging atlas. We subset the 672,765 T cells and ran the scVI data integration workflow, dimensionality reduction, and clustering workflow. We first performed cluster annotation for the CD4^+^ T cell populations and identified CD4^+^ Tn (*LRRN3*^+^, *CCR7*^hi^), Tcm (*LRRN3*^-^, *PASK*^+^), Tem (*KLRG1*^+^, *PASK*^lo^), and CD4^+^ cytotoxic lymphocytes (CD4 CTL; *GNLY*^hi^, *NKG7*^hi^). The CD4^+^ T cell population also contained T regulatory cells (Treg) with high *FOXP3*, *CTLA4*, and *IL2RA* expression (Fig. 3a, e). Among the CD8^+^ T cells, we identified CD8^+^ Tn (*LRRN3*^*+*^, *CCR7*^*hi*^, *PASK*^*hi*^), Tcm (*CCR7*^*lo*^, *PASK*^*lo*^), and Tem (*GNLY*^*hi*^, *NKG7*^*hi*^). We also annotated a population of gamma-delta T cells (Tgd; *TRDC*^*hi*^), mucosal-associated invariant T cells (MAIT; *SLC4A10*^*hi*^), and natural killer T cells (NKT; *CD3D*^*lo*^, *CD8*^*lo*^, *NCAM1*^*lo*^). In addition, we found a minor population of double-negative T cells (Tdn) that expressed neither *CD4* nor *CD8A*. Interestingly, we identified a distinct cluster of T cells (Tribo) marked by the enrichment of ribosomal genes (*RPL10, RPL8, RPL11 and RPS13)* as well as Ubiquitin Like and Ribosomal Protein S30 Fusion (*FAU*) gene (Fig. S7c). This T cell population was virtually absent in 3' datasets; the GSE213516 and HRA003766 datasets and was very minor in GSE214546 (0.14% of T cells).

We wanted to know how our manual annotations fared compared to the automated reference-based cell type classification. To this end, we utilized CellTypist software^15^ that implements regularized linear models coupled with a majority voting procedure to perform cell type predictions based on the manually curated reference datasets. Interestingly, the CellTypist predictions showed a high overlap between “Tcm/Naive helper T cells” and “Tcm/effector helper T cells” reference classes (Fig. S7d). Taken together, these results highlight the importance of manual marker inspection and validation, especially in the context of T cell annotation.

We next investigated whether the annotated T cell populations were equally represented in each dataset. Notably, the GSE214546 and syn22255433 data had more CD4^+^ Tn cells than other datasets (Fig. 3b, Table S2b). We also observed very few NKT and CD4^+^ CTL cells in the syn22255433, GSE157007, and GSE214546 datasets, while the other four were comparable in their proportions. As in the case of the total PBMCs, HRA000203 and HRA000624 were similar in their T cell subset proportions.

Similar to total PBMCs, we observed age and sex differences between the T subpopulations. In particular, the young group had more CD4^+^ Tn, CD8^+^ Tn, and MAIT cells but fewer CD8^+^ Tem cells (Fig. 3c). Interestingly, the female group had more CD8^+^ Tcm and CD8^+^ Tem cells (Fig. 3d).

### *MALAT1*-high T cells cluster separately and are highly heterogeneous

**Fig. 4:**
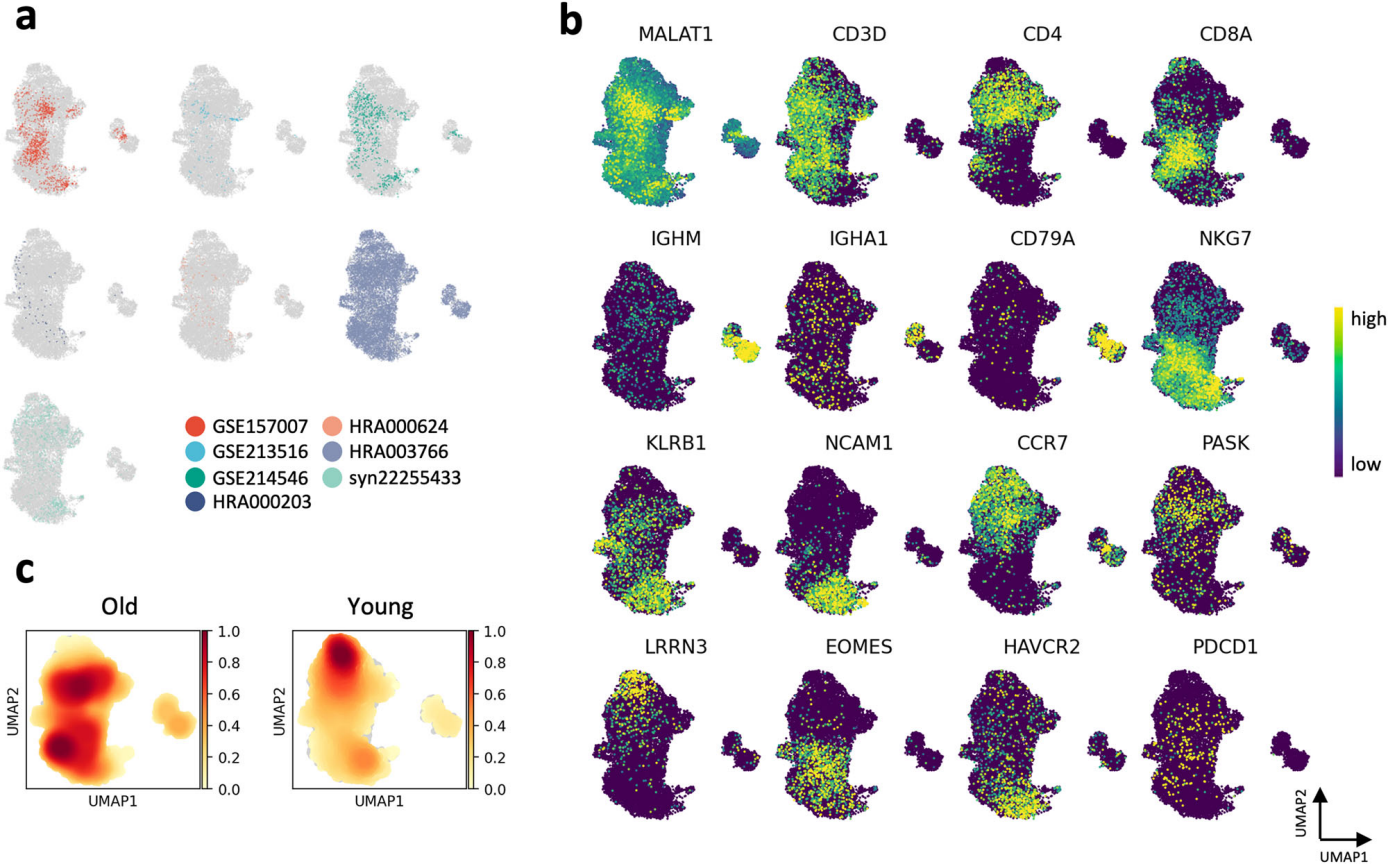
*MALAT1*^*hi*^ cells are highly heterogeneous. **a** UMAP representation of 14,007 *MALAT1*^*hi*^ cells colored by the dataset of origin. **b** Gene expression of selected marker genes. **c** Gaussian kernel density estimation of young and old groups density in the UMAP space.

Our integrated PBMC analysis showed a distinct *MALAT1*^*hi*^ cell population outside of major PBMC main T cell clusters (Figs. 2a, S3a, b). We focused on this relatively new T cell population because *MALAT1* has been proposed as a marker of exhausted T cells^1^ and epigenetic regulator of CD8^+^ terminal effector memory cell differentiation^16^. This abundant lncRNA is localized to the nucleus and differentially expressed in CD4^+^ and CD8^+^ T cells, with higher expression in Tregs and memory subsets^17^. After the batch effect correction and dimensionality reduction, we subset the *MALAT1*-high cluster (14,007 across all datasets) and examined its presence in all datasets (Fig. 4a). The HRA003766 contributed the majority of the cells (79.59%); other datasets had lower proportions of *MALAT1*^*hi*^ cells (GSE157007, 7.22%; syn22255433, 6.43%; GSE214546, 4.6%; HRA000624, 0.78%; HRA000203, 0.52%; GSE213516, 0.86%).

We used our T cell annotation markers to investigate the heterogeneity of the *MALAT1*^*hi*^ T cell community (Fig. 4b). In contrast to the main populations, the *MALAT1*^*hi*^ subset appeared highly heterogenous but had lower expression of *CD3D* (Fig. S3b). We found a distinct population of CD4^+^ T cells, which co-expressed the Tn markers *CCR7* and *LEF1*. Other T cells expressed *CD8A*, as well as *EOMES* and *NKG7*, which are expressed by terminal CD8^+^ Tem cells. We also observed a NK-like population with low *CD3D* expression but expressing cytotoxicity genes *NKG7, KLRB1*, and *NCAM1*. Surprisingly, we also observed a minor population of cells expressing markers of B cells. Together, this indicated that *MALAT1*^*hi*^ cells in our PBMC atlas contained both naïve-like and memory-like T cell subsets and cells of NK (or NKT lineage), as well as B cells. We next studied the abundance of *MALAT1*^*hi*^ cells between young and old groups (Fig. 4c). Interestingly, the *CD8A*^*+*^ cells were primarily present in old individuals, while the *CD4*^*+*^ cells originated from young donors, suggesting a differential expression of the *MALAT1* gene in young and old CD4^+^ and CD8^+^ T cell subsets.

### Abundance and transcriptome changes in aging

**Fig. 5:**
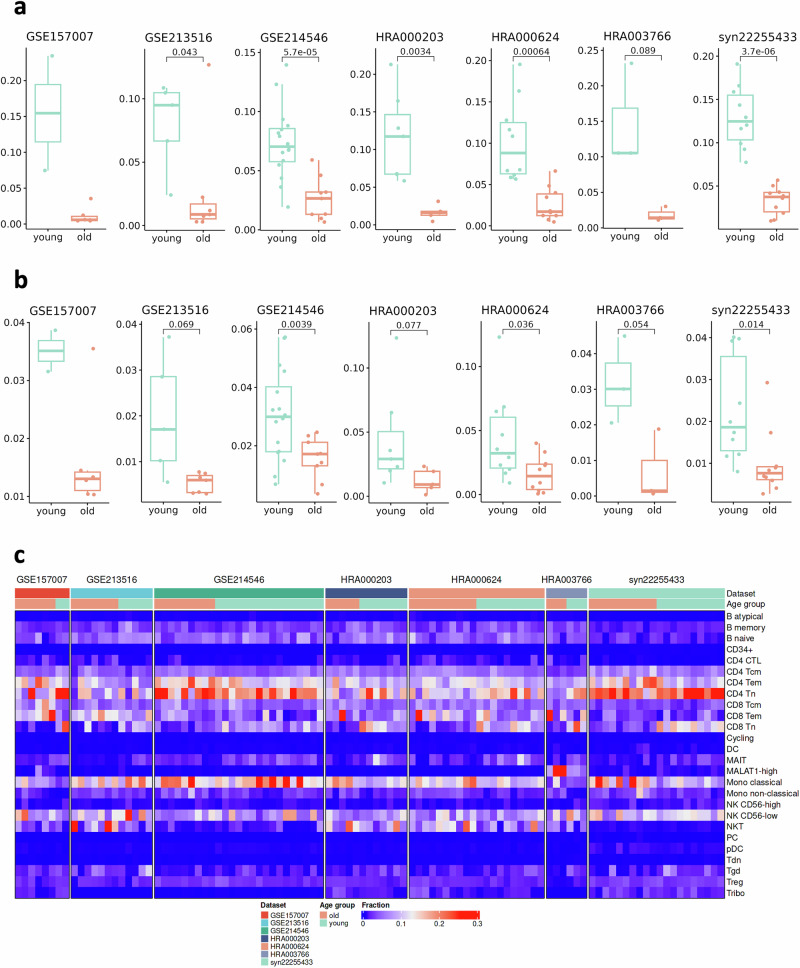
The cell type abundance differences in ageing. The proportion of (**a**) CD8 Tn and (**b**) MAIT cells in age groups and datasets. The numbers denote a p-value from a t-test. The test was not performed for GSE157007 due to the small young group size. **c** Heatmap of cell type proportion in each sample (columns are samples).

Using harmonized cell type annotations, we investigated the age-associated changes in the cellular abundance of each cell type across datasets. To this end, we performed a statistical test on the proportion of cells between young and old individuals in all datasets except GSE157007, which had only two samples in the young group. Our analysis reaffirmed the major alterations observed in immune cell aging. Notably, we saw a consistent reduction in CD8^+^ Tn in old in all seven datasets (Fig. 5a). The reduction was statistically significant in five datasets (GSE213516, GSE214546, HRA000203, HRA000624, and syn22255433; *p* < 0.05) and also present in GSE157007 dataset and HRA003766, although the difference in the latter did not reach statistical significance (*p* = 0.089). Also, CD4^+^ Tn was significantly lower in three datasets (HRA000203, HRA000624, HRA003766; *p* < 0.05, Figure S10d). In addition, we saw a consistent decrease of MAIT cells in old donors in all datasets with significant change in three datasets (GSE214546, HRA000624, and syn22255433; *p* < 0.05) (Fig. 5b). Non-classical monocytes were more prevalent in old donors compared to young samples in all datasets, although the effect was borderline significant in all datasets except HRA000624, where it reached statistical significance (Fig. S12b; *p* < 0.05). The other cell populations did not show consistent results (Figs. S9–15). Overall, we observed considerable variability in cell type prevalences among the datasets (Fig. 5c).

We next compared the gene expression in immune cells from young and old groups within each dataset to assess the age-related transcriptomic changes. We obtained sets of differentially expressed genes and associated log fold changes and performed their hierarchical clustering to assess whether the cell types in different datasets would cluster together. Strikingly, the clustering occurred mainly by dataset and not cell types, except for HRA000203 and HRA000624 (Fig. S8). These results indicate that the aging transcriptome differences were less pronounced than the variation between the datasets.

Next, we aimed to identify genes that are consistently differentially expressed with age across multiple datasets using a Wilcoxon signed-rank test. We analyzed each dataset separately to identify differentially expressed genes within each cell population (Table S3–S9). To obtain high-confidence gene lists and filter potential false positives, we searched for genes that were differentially expressed in at least four datasets. Focusing on the CD8^+^ Tn population, which was most consistently changed during aging, we obtained a gene signature with 43 genes most consistently upregulated in aged individuals (Table S16). We then performed an enrichment analysis and obtained GO:BP terms using g:Profiler^18^. Interestingly, the identified high-confidence driver terms included the “immune system process”(GO:0002376), “negative regulation of viral process”(GO:0048525), “response to external stimulus” (GO:0009605), and “cell activation” (GO:0001775), as well as “cell death” (GO:0008219).

Due to the susceptibility of the Wilcoxon signed-rank test to false positives, we validated the differential expression results using a pseudobulk approach (Tables S10–S15). Although this method yielded fewer hits across multiple cell types, the results for CD8^+^ Tn cells remained robust. The g:Profiler results included the “granzyme-mediated programmed cell death signaling pathway” (GO:0140507), “cell activation” (GO:0001775), “cell killing” (GO:0001906), “defense response” (GO:0006952), as well as “apoptotic process” (GO:0006915). Among the genes shared between the two types of DE analysis were genes associated with inflammation and effector function, such as *PTGER2*, *CLIC1*, *S100A4*, *PRF1*, *GZMA*, and *CCL5*. Collectively, these results demonstrated a consistent upregulation of multiple genes and related biological processes in CD8^+^ Tn cells during aging.

## Discussion

In this study, we built an integrated atlas of peripheral immune cells representing healthy human aging. While previous studies performed comprehensive analyses of transcriptomic changes in human PBMCs, inconsistent cell type annotations precluded their direct comparisons across different aging cohorts. Furthermore, our aging atlas enables the in-depth investigation of distinct cell populations due to the large number of cells included in our study. Our atlas comprises over a million annotated cells from 103 individuals. To our knowledge, this is the most extensive scRNA-seq re-analysis on the aging human immune system to date, and one of the largest datasets, together with the recent large-scale scRNA-seq profiling of ~2 million cells from healthy human blood at different ages^19^.

Our atlas enabled the detailed investigation of the *MALAT1*^*hi*^ population previously associated with T cell exhaustion aging^1^. *MALAT1* has recently been implicated in T cell development and associated with COVID-19 immune response^16,17,20^. Our analysis showed that *MALAT1*^*hi*^ cells were not equally distributed in seven datasets, with higher enrichment in GSE157007, HRA003766, and syn22255433. Interestingly, this cell population was heterogeneous in the expression of *CD3D* and T cell lineage markers (*CD4, CD8A*). Moreover, these cells varied considerably in their expression of naïve (*CCR7, PASK, LRRN3*) and exhaustion (*EOMES, PDCD1, HAVCR2*) markers. Taken together, our findings suggest that the *MALAT1*^*hi*^ cell population is highly heterogeneous. However, *MALAT1* is consistently the top expressed gene in 10x Genomics datasets, and its enrichment could suggest dying or damaged cells^21^. Recent analyses of *MALAT1* expression in scRNA-seq datasets suggest its potential utility as a quality control marker in the data processing workflow^22^. In line with this, we observed that the *MALAT1*^*hi*^ population in our data exhibited fewer UMIs per cell and a higher mitochondrial gene percentage than other PBMCs, indicating these cells may be under stress. Consequently, caution is warranted when interpreting the role of the *MALAT1*-enriched population in scRNA-seq data, as emerging evidence suggests it may serve as a quality measure in contrast to its biological relevance in T cell exhaustion.

We here propose the scRNA-seq annotation to distinguish Tn and Tcm subsets from PBMC data. Previously, Cano-Gamez and colleagues showed that *LRRN3* is expressed in CD4^+^ Tn but not CD4^+^ Tcm cells, while *PASK* is expressed at higher levels in CD4^+^ Tcm^23^. Our re-analysis of Tn and Tcm subsets demonstrated *LRRN3* as a subset marker not only for CD4^+^ but also CD8^+^ Tn cells, whereas the expression of PASK was specific for CD8^+^ Tn cells. In contrast, *CD69* was expressed at higher level in CD4^+^ and CD8^+^ Tcm populations. The employment of consistent annotation markers is critical for proper cell identification and the prerequisite for the comparative analysis of scRNA-seq datasets.

From all PBMC populations, the CD8^+^ Tn subset showed the most consistent change during aging, confirmed by our analysis of individual cohorts and the integrated dataset. The decrease in CD8^+^ Tn numbers has been associated with thymic involution, their increasingly defective regeneration capacity and conversion into effector memory phenotype cells over years^24,25^. Despite the high experimental variability, the scRNA-seq datasets shared multiple up- or downregulated genes in young and old individual samples. We found an increased expression of multiple effector and inflammation-associated genes in old individuals, including *PTGER2*, *CLIC1*, *S100A4*, *PRF1*, *GZMA*, and *CCL5*. We noted the upregulation of the same genes in a large dataset that studied the transcriptional landscape of age in PBMCs (*PTGER2* and *CLIC1*)^26^ and our transcriptome analysis on aging CD8^+^ T cells (*CLIC1* and *S100A4*)^27^. PTGER2 is a receptor for prostaglandin E2, a key mediator of inflammation. It has been shown to induce senescence markers, loss of CD28 expression, increased p16 cell cycle inhibitor expression, and diminished IL-2 and IFNγ production in CD8^+^ T cells^28^. The *CLIC1* gene encodes a chloride intracellular channel 1 and is a sensor of oxidative stress in cell nucleus. S100A4, is a potent trigger of inflammatory processes and is secreted in response to stress situations, stimulating the release of several cytokines and growth factors in the lymphoid and myeloid cells^29^. *PRF1* encodes perforin, a protein essential for the cytotoxic function of CD8^+^ T cells, enabling them to lyse target cells by forming pores in their membranes^30,31^. *GZMA* encodes granzyme A, a serine protease that, in addition to its role in cell killing, can stimulate the release of pro-inflammatory cytokines^32,33^. Finally, CCL5, also known as RANTES, is a chemokine critically involved in the inflammatory response by mediating the recruitment of leukocytes to sites of infection^34^. Thus, the upregulation of these genes may reflect the higher inflammatory environment in the blood of old individuals. Inflammageing, used to describe age-related increase in the levels of pro-inflammatory markers is considered a strong risk factor for multiple chronic age-related diseases^35^. Nevertheless, the functional role of these genes in naïve CD8^+^  T cell homeostatic proliferation, maintenance and priming capacity, and their pathophysiological correlations remain to be studied.

Despite collating an extensive collection of cells from many donors and multiple studies, comprehensive phenotype-metadata associations remain challenging. As the age group definitions vary between the studies, the results of cell type abundance comparisons and differential expression should be interpreted cautiously. Recent scRNA-seq studies have demonstrated cell type-specific effects of genetic variation on gene expression, which may contribute to the variability seen in our analysis^36^. Our study also included datasets from different ethnic origins. In addition, the metadata provided by the authors lacks standardization. For example, the HRA000624 metadata did not specify the exact age of donors but rather the age range. Factors like genetic background, ethnicity and socioeconomic status could influence immune phenotypes, highlighting the importance of collecting additional metadata for the analysis.

In conclusion, we conducted an extensive re-analysis and cell type annotation of seven PBMC aging datasets, revealing significant disparities in cell abundance across different annotation levels. Our analysis identified pro-inflammatory changes in CD8^+^ Tn cells, alongside a consistent reduction in their numbers in elderly individuals. Additionally, we found that *MALAT1*^*hi*^ cells are heterogeneous, likely comprising a mix of cells with naïve-like and exhausted-like phenotypes. Collectively, our aging atlas serves as a valuable reference, laying the groundwork for a deeper understanding of human immune aging.

## Methods

### Data acquisition

We performed a comprehensive literature review and selected seven scRNA seq studies involving PBMCs in old and young age. The gene expression matrices from the *Luo* et al., Zhu et al., and Thomson et al. studies were downloaded from the Gene Expression Omnibus (accessions GSE157007, GSE213516, and GSE214546). The raw sequencing data from Zheng et al., Hung et al., and Li et al. were obtained from the National Genomics Data Center (accessions HRA000203, HRA000624, and HRA003766). The Mogilenko et al. raw data files were downloaded from Synapse (accession syn22255433). The GSE213516, GSE214546, and HRA003766 datasets were generated using the 3' version of the 10x protocol, while the rest were processed with the 5' kits.

### Raw data processing and quality control

The CellRanger pipeline from 10x Genomics was used to align the reads and generate feature-barcode matrices for the HRA000203, HRA000204, HRA003766, and syn22255433 data. We downloaded the pre-built GRCh38 human reference from 10x Genomics for transcriptome mapping. After obtaining the gene expression matrices for all seven studies, the data were loaded into Scanpy^37^. We first used the UMI counts and the percentage of mitochondrial gene expression to exclude the low-quality cells. We performed QC for each sample in each dataset individually, as the samples were processed as standalone 10x libraries, and we reasoned that the quality thresholds should be independent of the samples. To select the appropriate QC thresholds, we first assumed that most of the cells in the samples were of good quality. We then manually selected the appropriate thresholds for each sample to exclude most outliers (Fig. S1a–g). When the UMI count distribution was bimodal, we assumed that both low- and high-quality cells were present in the sample. In such a case, we aimed to exclude most of the cells in the low-quality tail of the distribution.

In addition, the cells with high levels of *CMTM5, ITGA2B, HBA1*, and *PF4* and low levels of *PTPRC* expression were removed to exclude non-immune cells. We have also removed genes coding for immunoglobulin chains and T cell receptors to avoid spurious clustering of B and T cells based on biased retention of V(D)J transcipts in the 5' gene expression libraries. The resulting filtered gene expression matrices were combined into one AnnData object and used for the downstream analyses.

### Doublet removal

To identify doublets, we applied the scDblFinder R package^38^. To account for differences between random initializations, we marked a cell as a potential doublet if scDblFinder classified the cell as a doublet in at least 6 out of 10 runs. This classification was subsequently used during the UMAP and clustering analysis to identify cell neighborhoods enriched in putative doublets. After visually inspecting the UMAP with the overlayed doublet calls, and confirmed that putative doublets were forming distinct communities. Clusters enriched in doublets were removed, and the batch effect correction workflow was repeated.

### Batch effect correction and dimensionality reduction

The scVI package^10^ was used to remove the batch effect between the samples and the datasets. First, the top 5000 highly-variable genes were selected within each dataset and then merged as previously described using Scanpy. Next, an scVI model was created, and unnormalized gene expression values were used as inputs. The dataset and donor identifier were added to the model as categorical covariates. The mitochondrial gene expression percentage in each cell was used as a continuous covariate to account for the unwanted technical variation. The final model was trained for 400 epochs which produced a 10-dimensional latent data representation for each cell. The latter was used as the input for the downstream analysis.

The UMAP representation and Leiden clustering were obtained using the Scanpy package. In brief, a neighborhood graph was constructed using the scVI latent space as input. Next, the UMAP representation was built using the umap-learn implementation through Scanpy. Leiden clustering was performed at different resolutions and clusters were annotated based on the expression of canonical marker genes.

We used a Python implementation of the Harmony algorithm^11^ in the alternative integration workflow. To this end, we called the batch effect correction function available in the Scanpy package. The Harmony PCA space was used to compute the neighborhood graph and UMAP embedding. The clustering was performed as described in the scVI workflow.

### CellTypist analysis

To annotate the T cell subsets with CellTypist^15^, we first downloaded the “Immune_All_Low” model trained on the immune sub-populations combined from 20 tissues of 18 studies and contains almost a hundred cell types. Next, we imported the combined gene expression matrix for the T cells from all the datasets and performed the library size normalization and log scaling. Next, we obtained the CellTypist predictions using the majority voting procedure.

### Differential expression analysis

To conduct the DE test analysis, we used the Wilcoxon rank-sum test implemented in the Scanpy package. We excluded cell types that had fewer than thirty cells in either the old or young group. To obtain the high-confidence results, we set the significance threshold at 0.25 for log-fold change and 0.05 for FDR. In order to perform the hierarchical clustering of using the DE results, we calculated the distance matrix using Euclidean measure. This matrix was used as an input to the complete linkage method implemented in the *stats* package.

We employed the pseudobulk approach implemented in the glmGamPoi R package to validate DE results^39^. To this end, we first constructed pseudobulk profiles for each cell type and sample combination while excluding profiles with less than ten cells. The DE tests were performed for cell type within the dataset if there were at least three pseudobulk profiles in the age group. To identify the biological processes enriched in the DE results, we used the g:Profiler tool^18^. The two-stage algorithm implemented in g:Profiler to identify driver GO terms was used to reduce redundancy in GO enrichment results.

## Supplementary information

Supplementary figures

Table S1

Table S2

Table S3

Table S4

Table S5

Table S6

Table S7

Table S8

Table S9

Table S10

Table S11

Table S12

Table S13

Table S14

Table S15

Table S16

## Data Availability

All data used in the study are available under corresponding accessions on NCBI GEO (GSE157007, GSE213516, GSE214546), NGDC GSA (HRA000203, HRA000624, HRA003766), and Synapse (syn22255433).

## Code availability

Analytical scripts and codes are available at https://github.com/f6v/PBMC_ageing_atlas.
