# Supplementary material for: An integrated single-cell atlas of blood immune cells in aging
Source: NPJ Aging. 2024 Nov 29;10(1):59. doi: 10.1038/s41514-024-00185-x (PMC11606963; doi:10.1038/s41514-024-00185-x)
Supplement: Supplementary file 1 — Supplementary figures [file 41514_2024_185_MOESM1_ESM.pdf]

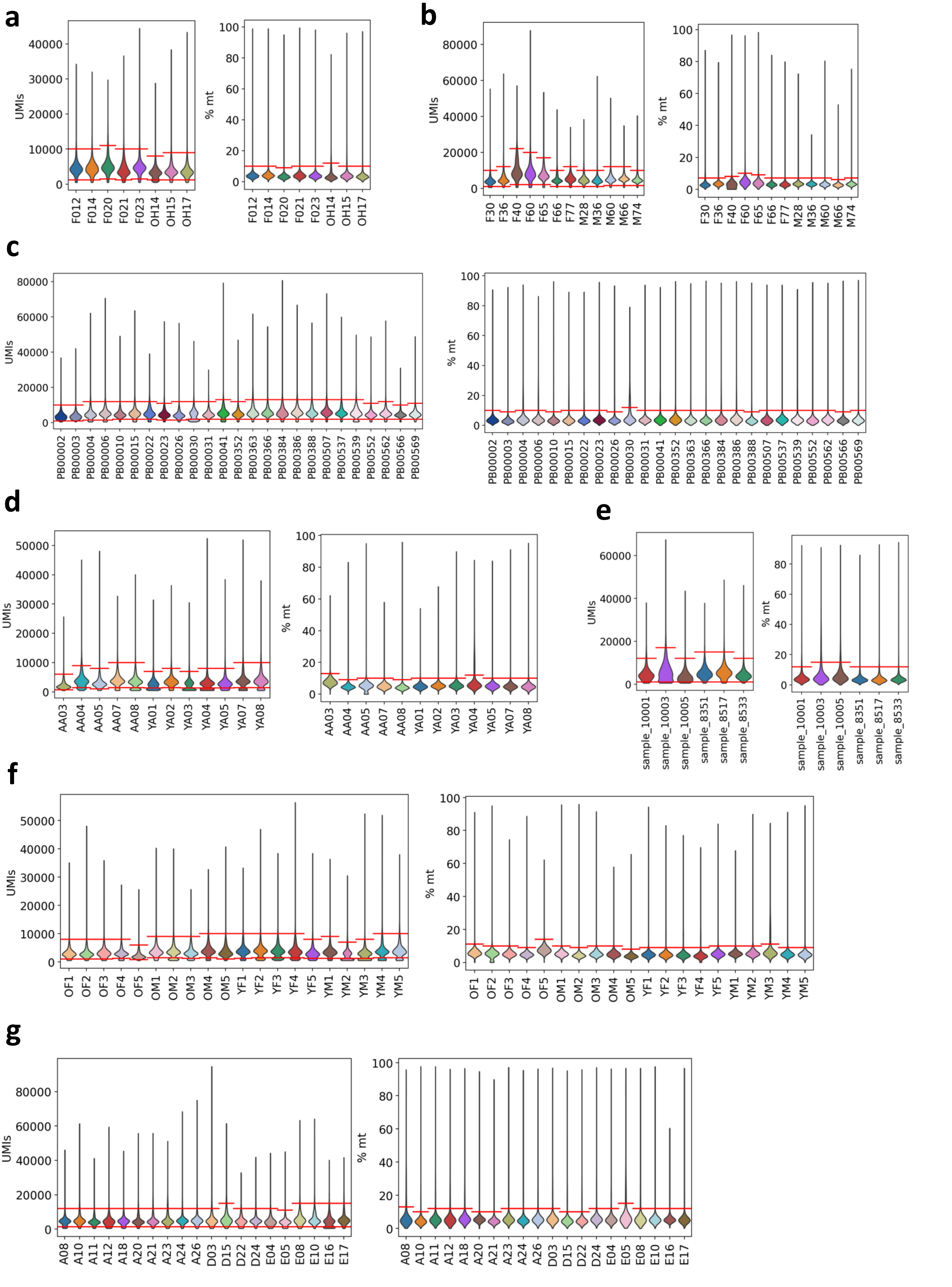

Supplementary figure S1. Quality control metrics. The number of UMIs/cell and mitochondrial gene counts percentages for each sample in **a** GSE157007, **b** GSE213516, **c** GSE214546, **d** HRA000203, **e** HRA003766, **f** HRA000624, and **g** syn22255433. The red lines represent the selected threshold for each sample.

a

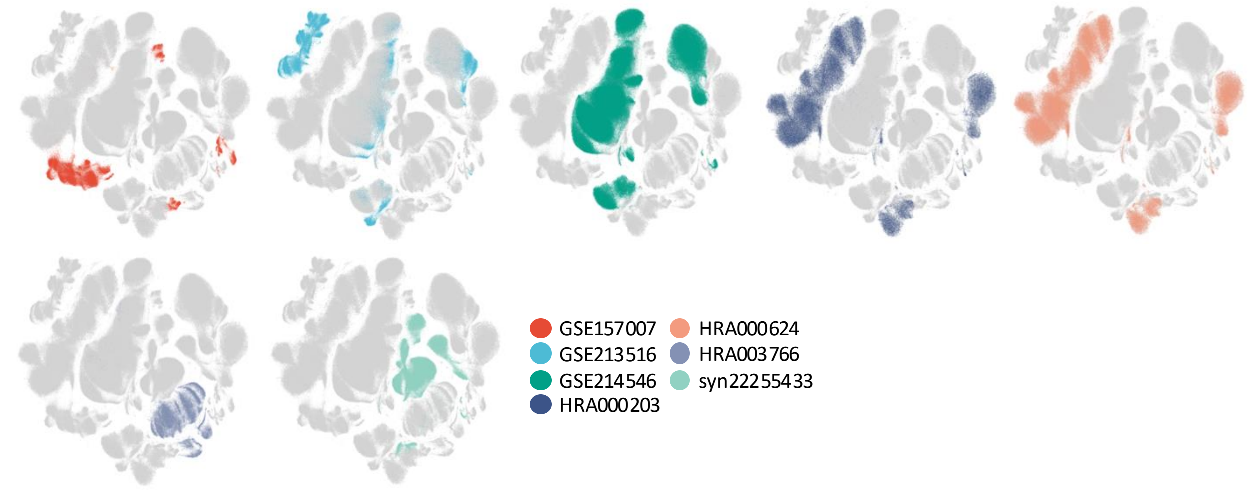

b

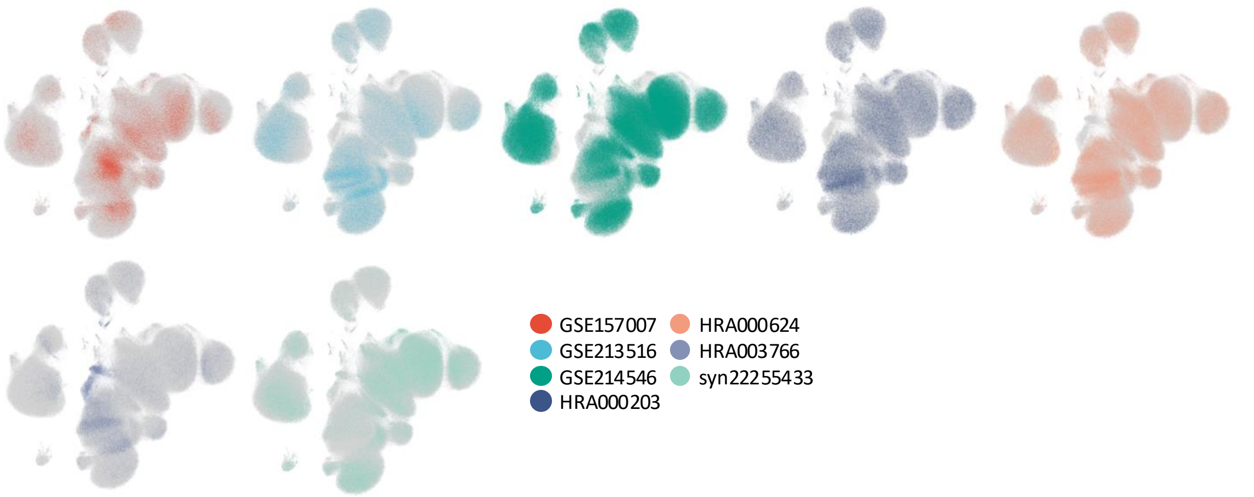

c

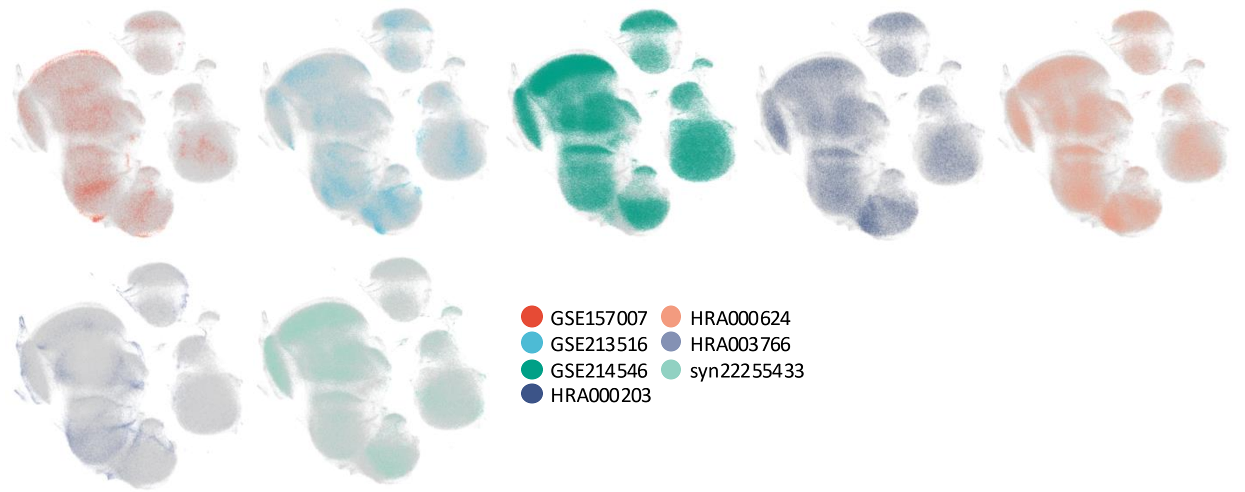

Supplementary figure S2. Batch effect correction ameliorates technical differences. a The UMAP representation of seven ageing PBMC datasets before the batch effect correction, **b** with scVI integration, and **c** with Harmony integration.

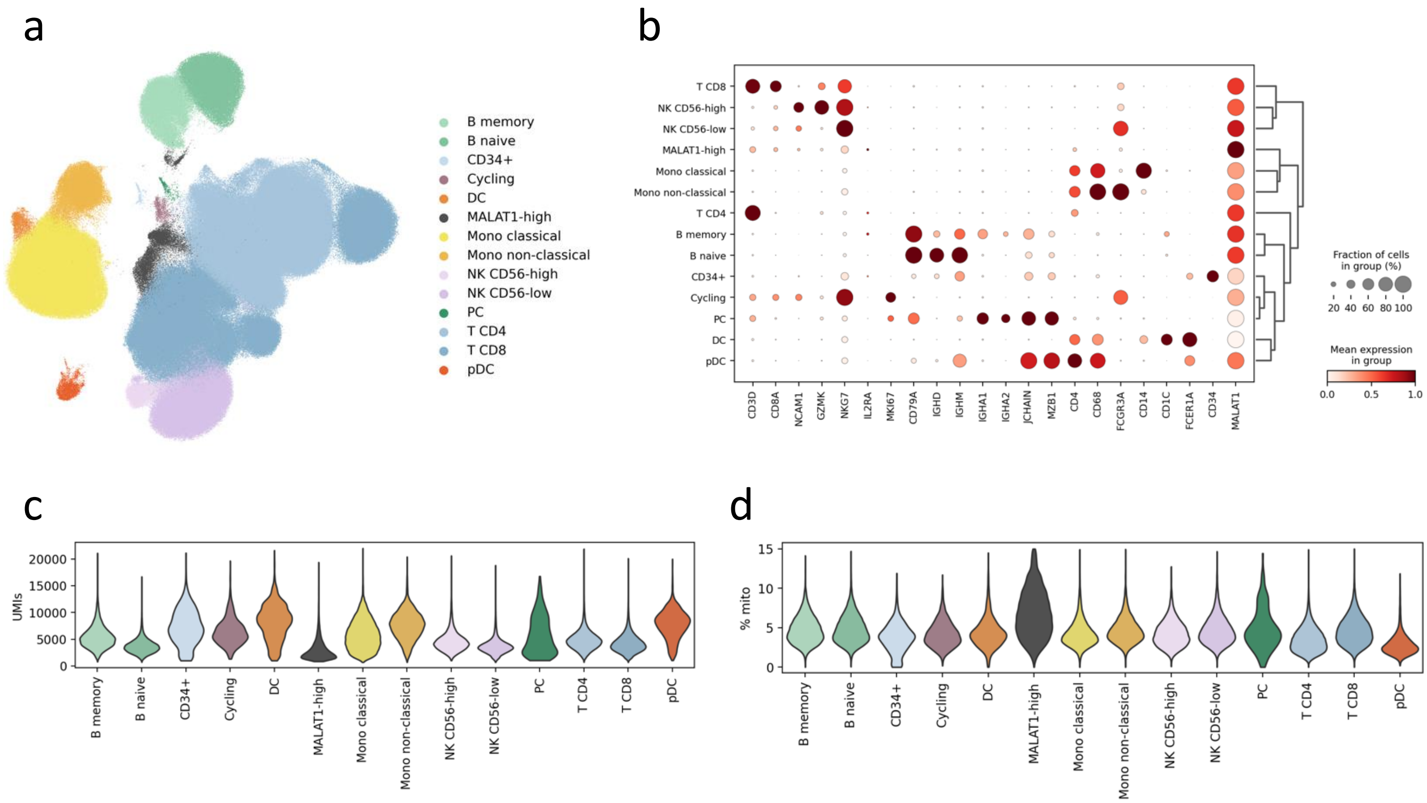

Supplementary figure S3. scVI-integrated PBMC ageing atlas. a UMAP representation of major PBMC cell types identified by integrating seven ageing datasets. **b** Dotplot with marker genes for major PBMC cell types. **c** The distributions of UMIs per cell and **d** mitochondrial gene expression percentage per cell in the annotated cell types.

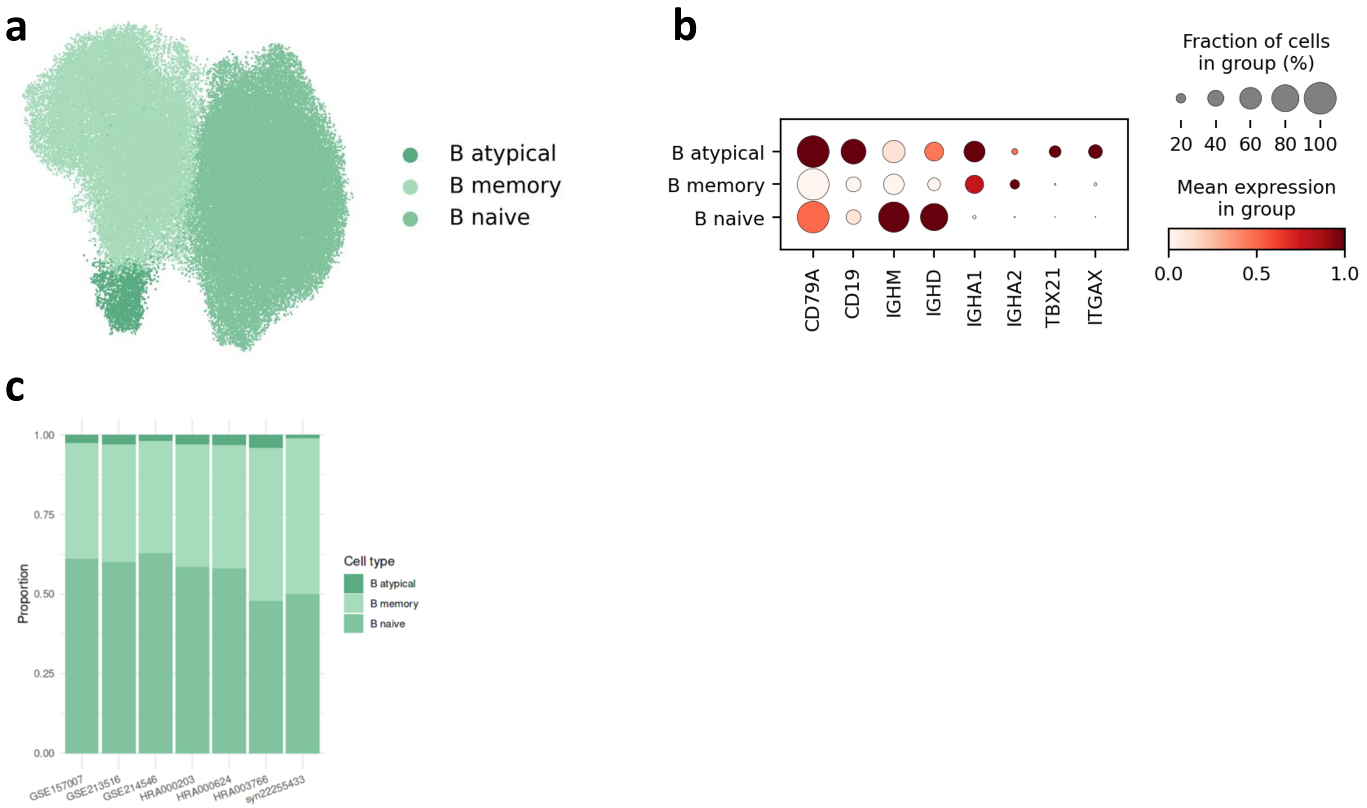

Supplementary figure S4. Integrated analysis of young and old B cells. a UMAP representation of the 77,378 B cells from young and old subjects identified by reanalysis of B cell subsets. **b** Dotplot of selected marker genes used to identify the B cell subsets. **c** Bar plots showing proportions of B subpopulations in each dataset.

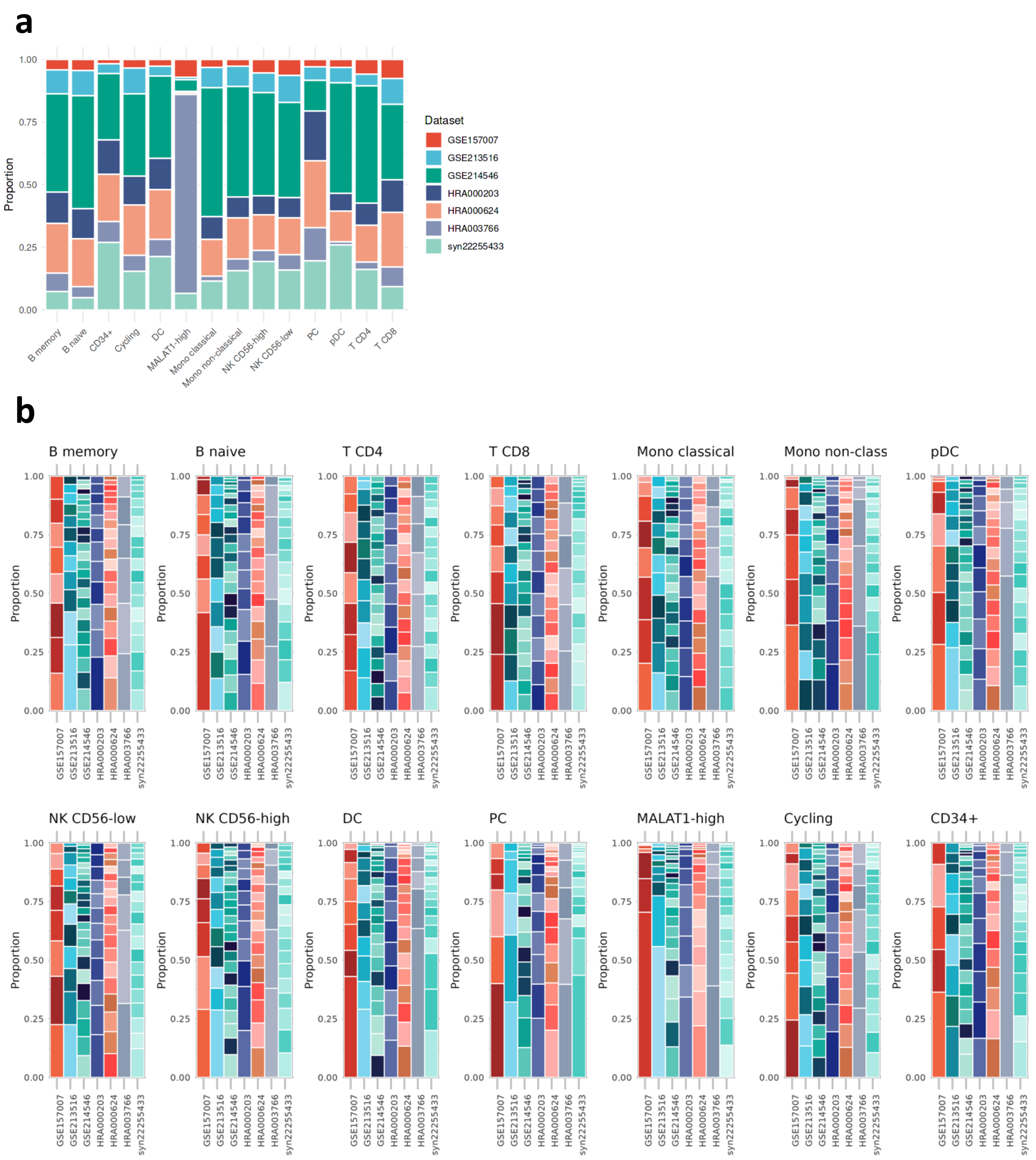

Supplementary figure S5. The contribution of datasets and samples. a The proportion of cells from each dataset in PBMC cell types. **b** Each bar within the dataset (columns) represents the proportion of cells in a cell type originating from a unique sample.

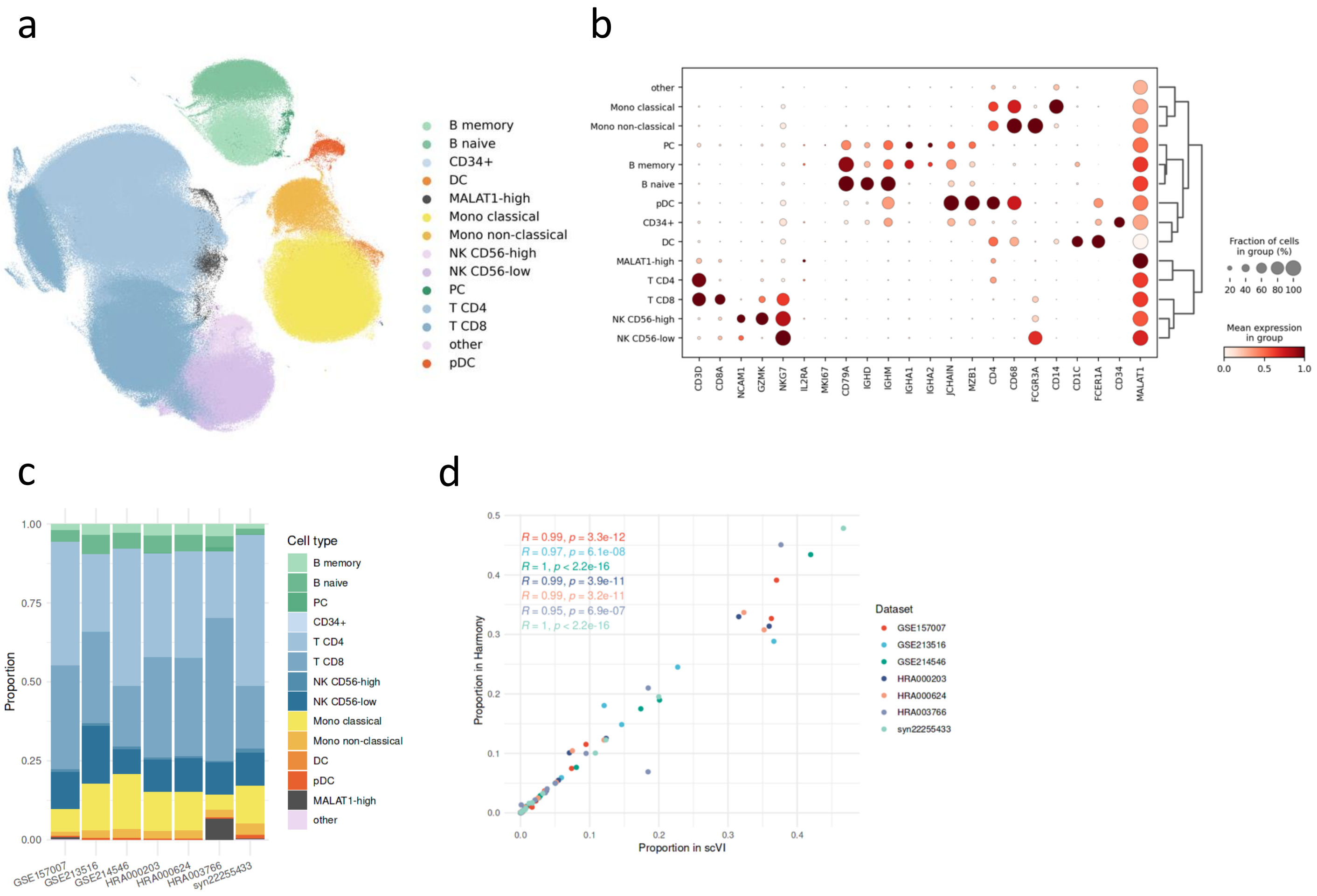

Supplementary figure S6. Alternative integration using Harmony. a UMAP representation of major PBMC cell types identified by integrating seven ageing datasets. **b** Dotplot with marker genes for major PBMC cell types. **c** Bar plots showing proportions of the major PBMC subpopulations in each dataset. **d** Correlation between the cell type proportions in scVI and Harmony results. Each dot represents a cell type in a dataset.

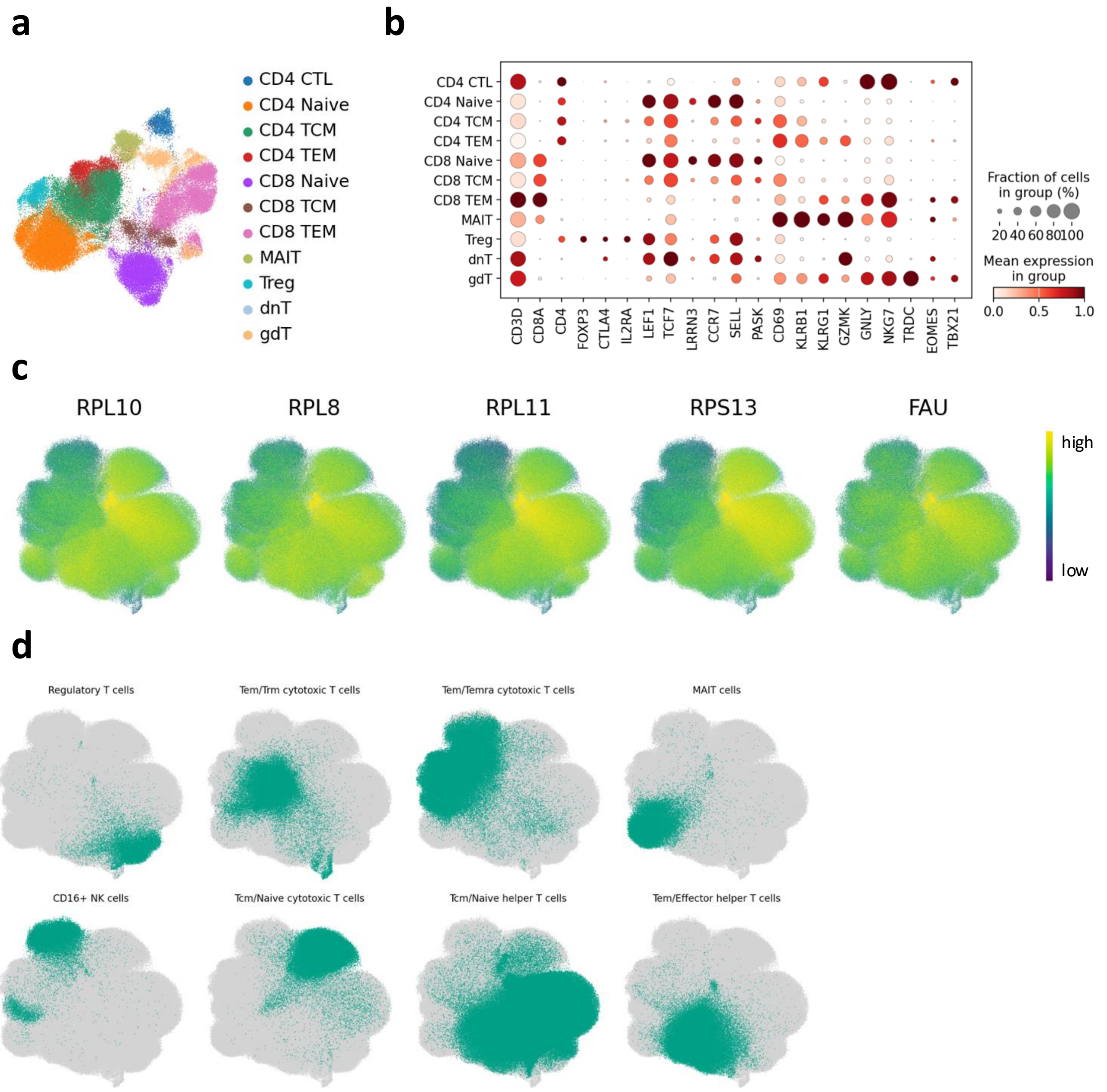

Supplementary figure S7. T cell subtypes annotation. a UMAP of T cells from the reference CITE-seq dataset used for T marker validation. **b** Reference T subset marker genes. **c** UMAP representation of the T cells from young and old subjects with the gene expression levels of the Tribo subset markers. **d** UMAP representation of the T cells from young and old subjects with the CellTypist predictions. The cells designated to a specific class are depicted by green dots, while all remaining cells are represented by grey dots.

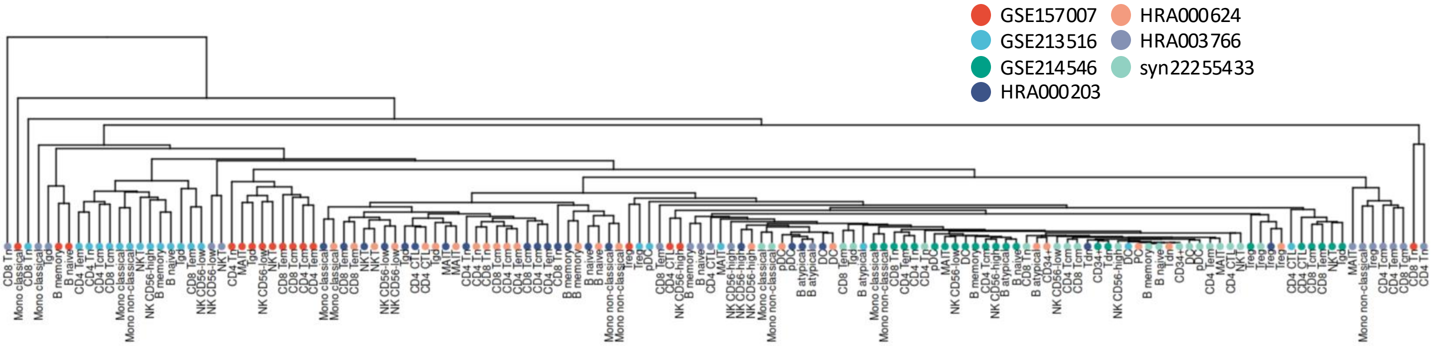

Supplementary figure S8. Characterization of differential expression similarity between the datasets. The LFC values from aging DE in each cell type and dataset were concatenated and hierarchically clustered. The colors of the circles on the tree leaves represent the dataset of origin.

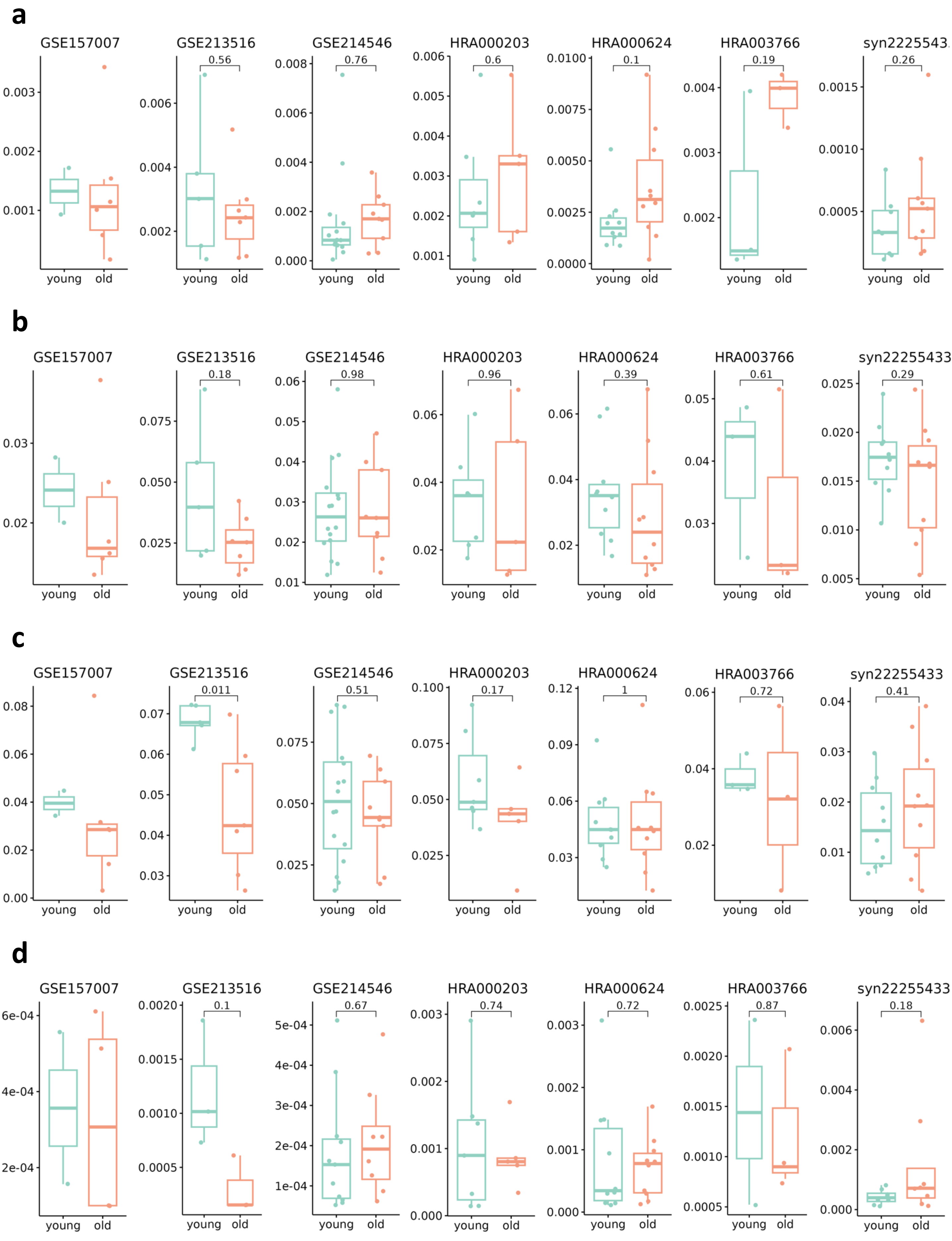

Supplementary figure S9. Cell type abundance comparison between young and old groups in **a** B atypical, **b** B memory, **c** B naïve, and **d** PC. The numbers denote a p-value from a t-test. The test was not performed for GSE157007 due to the small young group size.

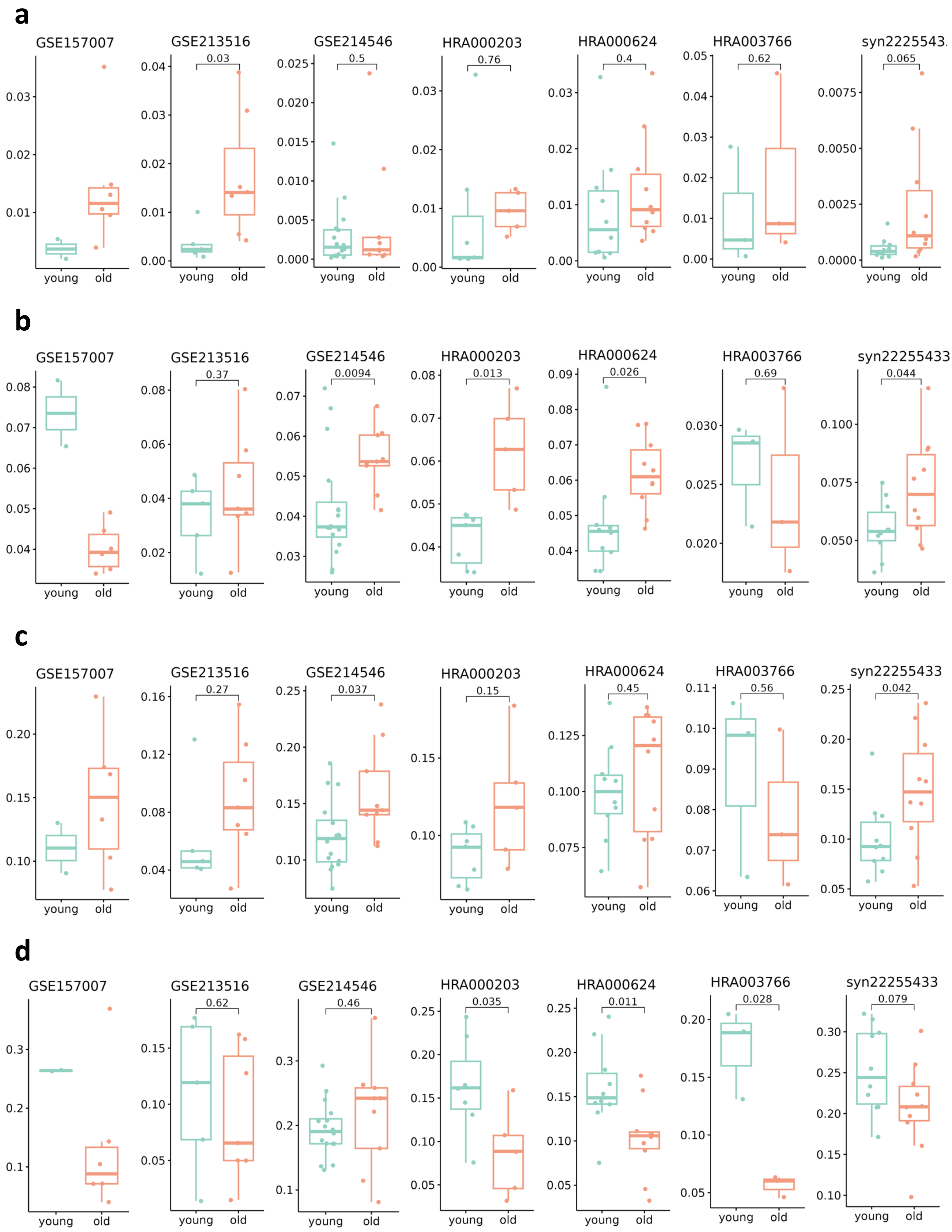

Supplementary figure S10. Cell type abundance comparison between young and old groups in **a** CD4 CTL, **b** CD4 Tcm, **c** CD4 Tem, and **d** CD4 Tn. The numbers denote a p-value from a t-test. The test was not performed for GSE157007 due to the small young group size.

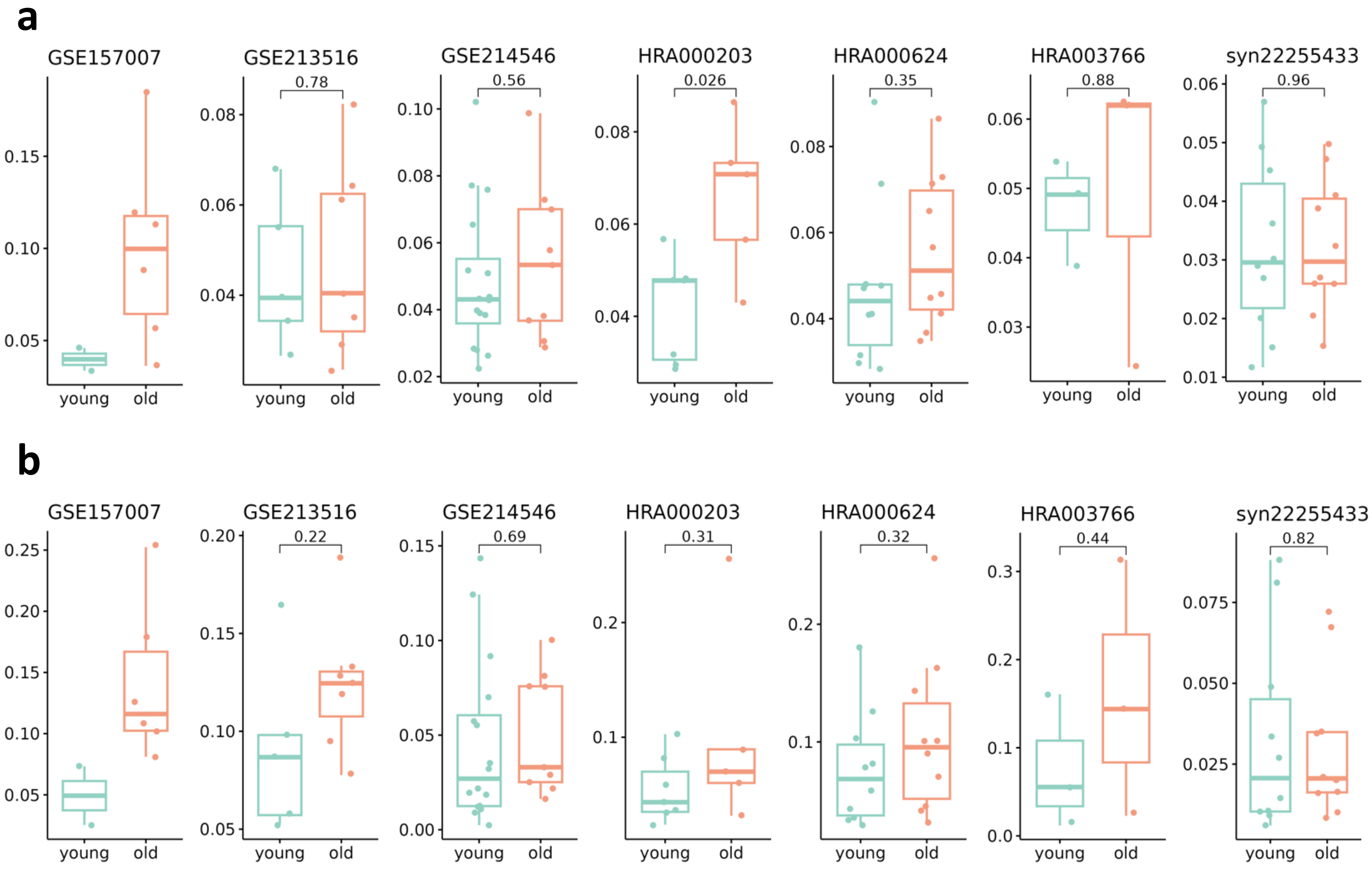

Supplementary figure S11. Cell type abundance comparison between young and old groups in **a** CD8 Tcm and **b** CD8 Tem. The numbers denote a p-value from a t-test. The test was not performed for GSE157007 due to the small young group size.

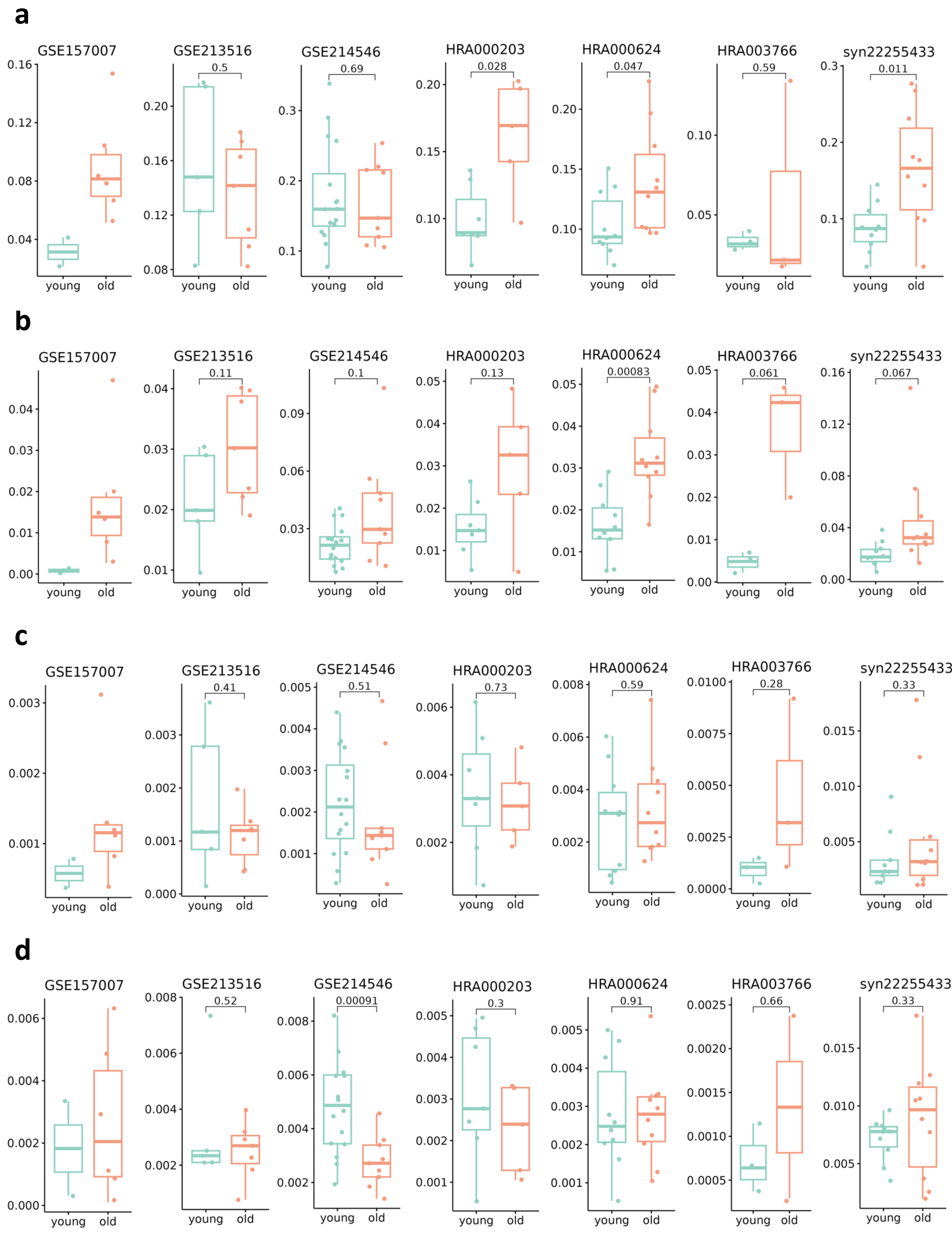

Supplementary figure S12. Cell type abundance comparison between young and old groups in **a** Mono classical, **b** Mono non-classical, **c** DC, and **d** pDC. The numbers denote a p-value from a t-test. The test was not performed for GSE157007 due to the small young group size.

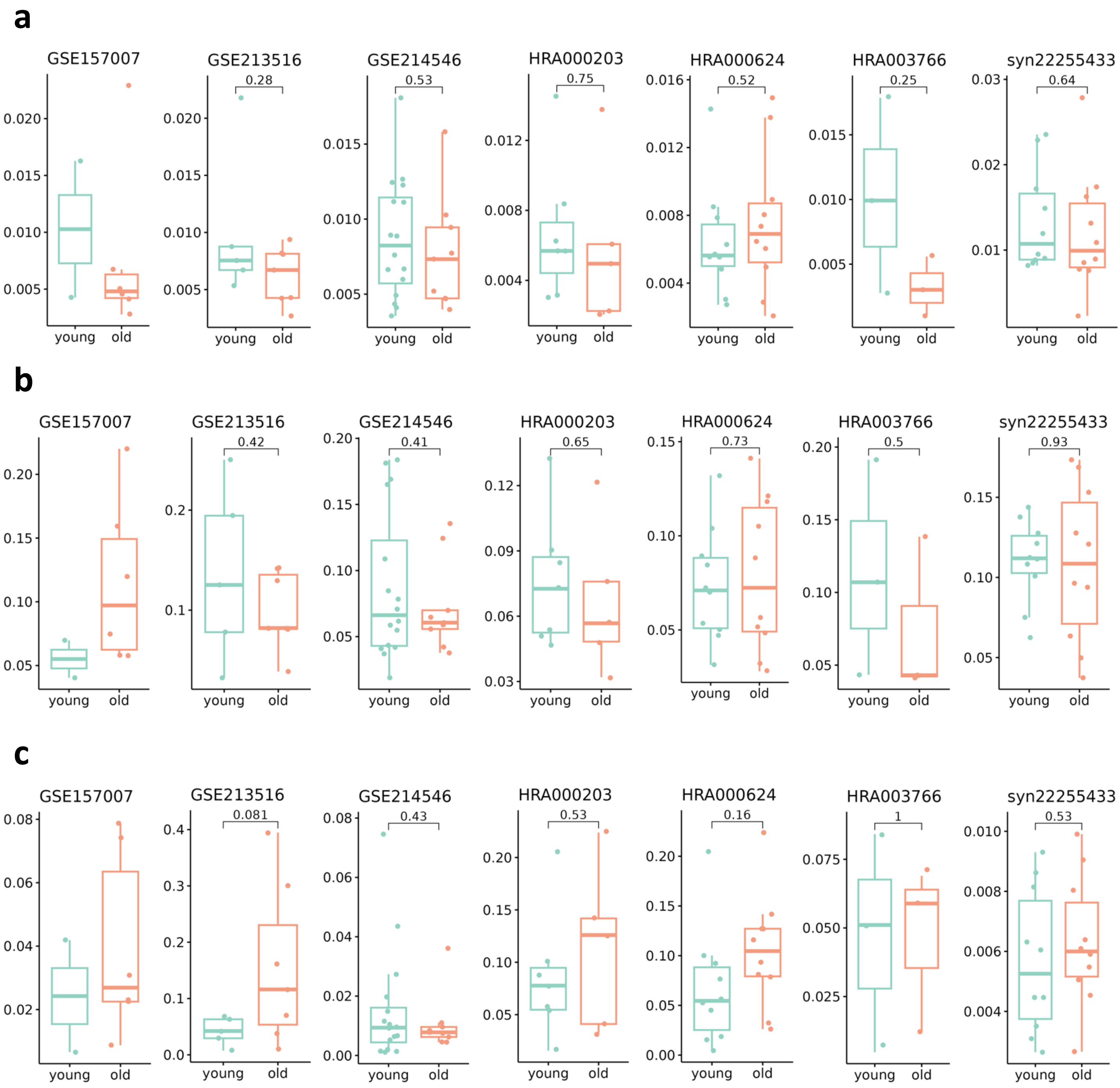

Supplementary figure S13. Cell type abundance comparison between young and old groups in **a** NK CD56-high, **b** NK CD56-low, and **c** NKT. The numbers denote a p-value from a t-test. The test was not performed for GSE157007 due to the small young group size.

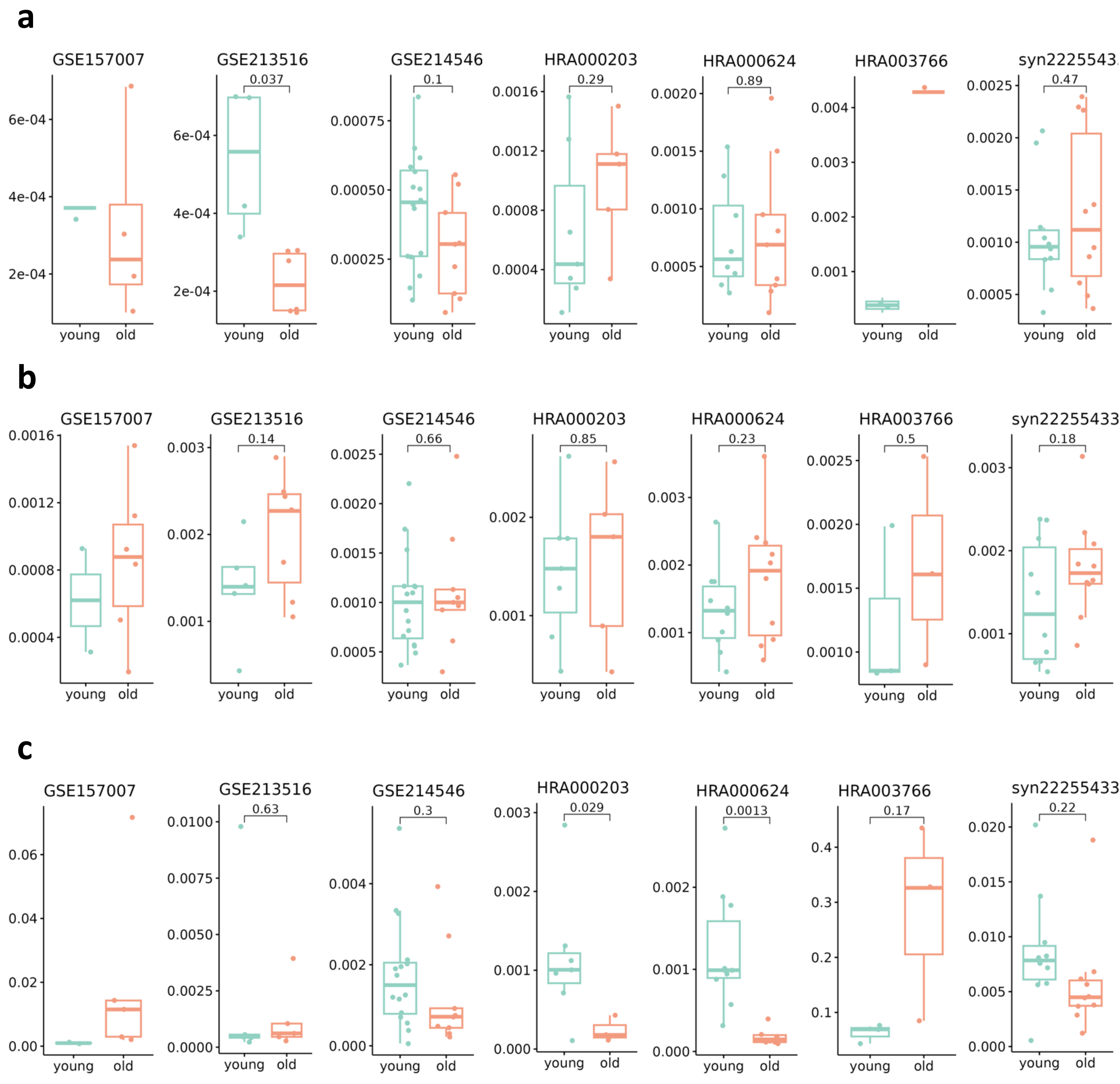

Supplementary figure S14. Cell type abundance comparison between young and old groups in **a** CD34+, **b** Cycling, and **c** MALAT1-high. The numbers denote a p-value from a t-test. The test was not performed for GSE157007 due to the small young group size.

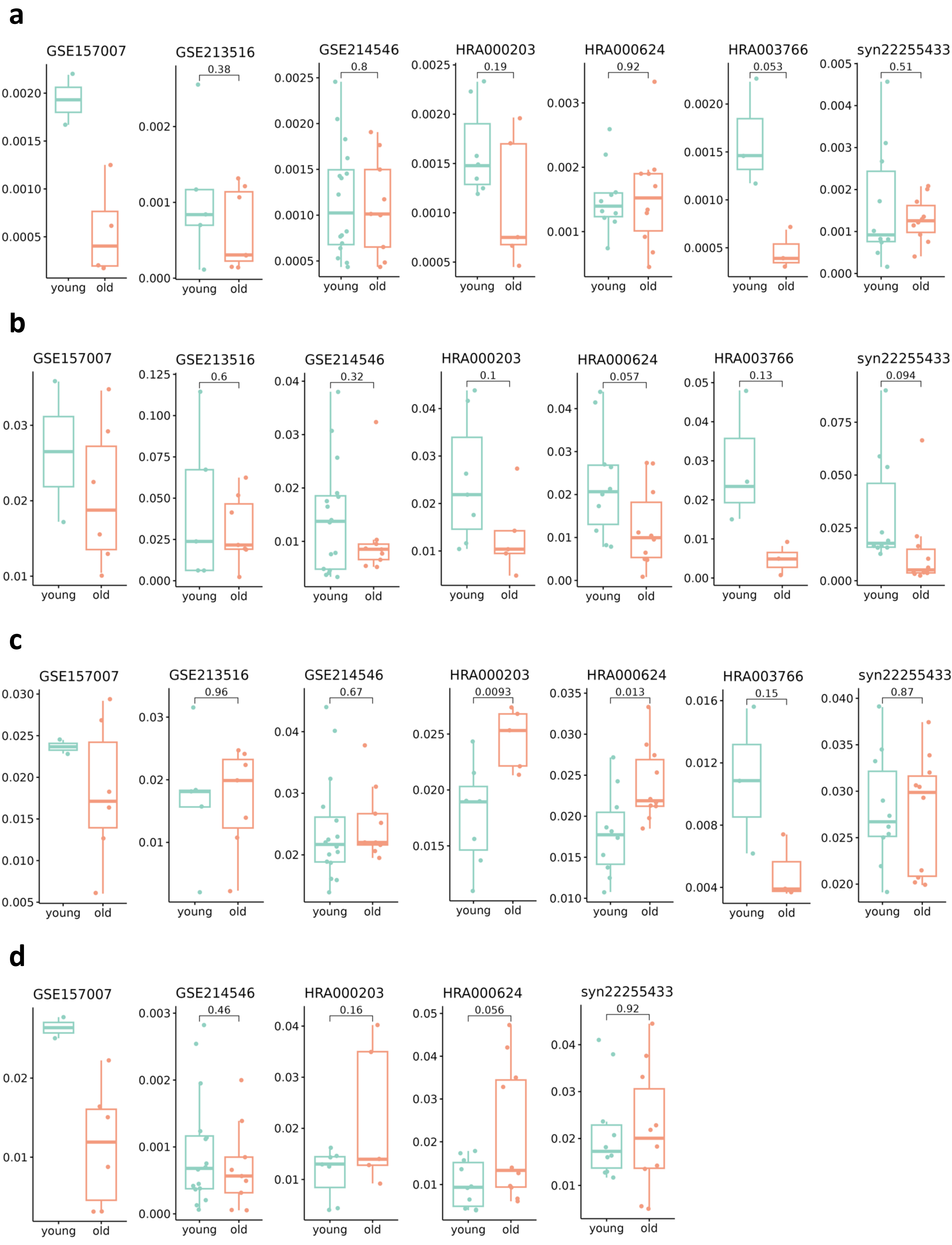

Supplementary figure S15. Cell type abundance comparison between young and old groups in **a** Tdn, **b** Tgd, **c** Treg, and **d** Tribo. The numbers denote a p-value from a t-test. The test was not performed for GSE157007 due to the small young group size.
